# Is Response to Fire Influenced by Dietary Specialization and Mobility? A Comparative Study with Multiple Animal Assemblages

**DOI:** 10.1371/journal.pone.0088224

**Published:** 2014-02-07

**Authors:** Xavier Santos, Eduardo Mateos, Vicenç Bros, Lluís Brotons, Eva De Mas, Joan A. Herraiz, Sergi Herrando, Àngel Miño, Josep M. Olmo-Vidal, Javier Quesada, Jordi Ribes, Santiago Sabaté, Teresa Sauras-Yera, Antoni Serra, V. Ramón Vallejo, Amador Viñolas

**Affiliations:** 1 CIBIO/InBIO (Centro de Investigação em Biodiversidade e Recursos Genéticos), Universidade do Porto, Vairão, Portugal; 2 Departament de Biologia Animal, Universitat de Barcelona, Barcelona, Spain; 3 Parc Natural de Sant Llorenç del Munt i l’Obac, Oficina Tècnica de Parcs Naturals, Diputació de Barcelona, Barcelona, Spain; 4 Grup d’Ecologia del Paisatge, Àrea de Biodiversitat, CEMFOR-CTFC (Centre Tecnològic Forestal de Catalunya), Solsona, Spain; 5 CREAF(Centre de Recerca Ecològica i Aplicacions Forestals), Universitat Autònoma de Barcelona, Bellaterra, Spain; 6 Departamento de Ecología Evolutiva, de la Conducta y Conservación, Estación Experimental de Zonas Áridas, CSIC (Consejo Superior de Investigaciones Científicas), Almería, Spain; 7 AIM (Asociación Ibérica de Mirmecologia), Facultat de Ciències, Universitat de Girona, Girona, Spain; 8 ICO (Institut Català d’Ornitologia), Museu de Ciències Naturals de Barcelona, Barcelona, Spain; 9 Servei de Biodiversitat i Protecció dels Animals, Direcció General del Medi Natural i Biodiversitat, Barcelona, Spain; 10 Museu de Ciències Naturals de Barcelona, Barcelona, Spain; 11 Departament d’Ecologia, Universitat de Barcelona, Barcelona, Spain; 12 Departament de Biologia Vegetal, Universitat de Barcelona, Barcelona, Spain; 13 Fundación CEAM. Parque Tecnológico, Paterna, Spain; University of Western Ontario, Canada

## Abstract

Fire is a major agent involved in landscape transformation and an indirect cause of changes in species composition. Responses to fire may vary greatly depending on life histories and functional traits of species. We have examined the taxonomic and functional responses to fire of eight taxonomic animal groups displaying a gradient of dietary and mobility patterns: Gastropoda, Heteroptera, Formicidae, Coleoptera, Araneae, Orthoptera, Reptilia and Aves. The fieldwork was conducted in a Mediterranean protected area on 3 sites (one unburnt and two burnt with different postfire management practices) with five replicates per site. We collected information from 4606 specimens from 274 animal species. Similarity in species composition and abundance between areas was measured by the Bray-Curtis index and ANOSIM, and comparisons between animal and plant responses by Mantel tests. We analyze whether groups with the highest percentage of omnivorous species, these species being more generalist in their dietary habits, show weak responses to fire (i.e. more similarity between burnt and unburnt areas), and independent responses to changes in vegetation. We also explore how mobility, i.e. dispersal ability, influences responses to fire. Our results demonstrate that differences in species composition and abundance between burnt and unburnt areas differed among groups. We found a tendency towards presenting lower differences between areas for groups with higher percentages of omnivorous species. Moreover, taxa with a higher percentage of omnivorous species had significantly more independent responses of changes in vegetation. High- (e.g. Aves) and low-mobility (e.g. Gastropoda) groups had the strongest responses to fire (higher R scores of the ANOSIM); however, we failed to find a significant general pattern with all the groups according to their mobility. Our results partially support the idea that functional traits underlie the response of organisms to environmental changes caused by fire.

## Introduction

Wildfires are natural disturbances that have shaped vegetation composition and structure, and influenced the associated faunas in almost all regions of the world [1]. In the Mediterranean Basin, characterized by a long, dry period with irregular rainfall, the combination of climatic and current anthropogenic effects have defined fire as a common disturbance [2]. Consequently, both natural and human-induced fires have modelled the landscape and are a fundamental element to understand Mediterranean ecosystem functioning and structure [3].

Over the short term, fire may act as an environmental filter that selects species better adapted to the narrow postfire environmental conditions [4]. Early postfire succession increases open areas and favours a shift in dominant species, often leading to different animal assemblages in burnt compared with unburnt areas [5]–[6], [7]–[8], [9]–[10]. Knowledge on how species respond to fire is a challenge, since responses vary greatly depending on particular life histories of each species [11]–[12], [13]. Some taxonomic groups undergo a postfire increase in the number of species [8]–[14], other groups show a reduction [9], [15]–[16], and still others display specific differences within the group (e.g. amphibians [17], reptiles [18]–[19], arthropods [2], birds [20]).

The response of animal communities to fire is driven primarily by habitat characteristics, i.e. vegetation structure and composition [21], [22]–[23]. Several studies have documented that animal communities respond to fire following habitat changes over postfire succession (e.g. [9] for snails, [18] for reptiles, [24] for birds]. These examples support the habitat-accommodation model of succession (but see [25]), which states that species enter a community when their preferred habitat type has developed and then decline as the plant succession proceeds beyond their optimal habitat conditions [26].

Specific functional traits have proved an excellent approach for understanding the mechanisms of community responses to fire [13]–[27]. Disturbances such as fire are processes which alter the niche opportunities available to the species in a system, causing shifts in available resources, and inflicting impacts on the niche relationships of the organisms [28]. Thus, the response of organisms to fire may be mediated by their ecological specialization in resource use. In a general way, the disturbance theory states that specialist species are negatively affected by disturbance, while generalist species benefit from it (e.g. [29]–[30]). In stable environments, generalists cannot outperform specialists due to the inherent extra physiological and behavioural costs associated with generalists, which accommodate multiple prey types, variable environments, or activity timing [31]. In variable environments, however, these costs may be small in comparison to the benefits inherent to the increased plasticity, and therefore generalists may gain an advantage with respect to specialists [30]. Hence, ecosystems characterized by abrupt environmental changes triggered by disturbances would promote generalist species [29].

In our study, we have examined the taxonomic and functional response to fire by eight terrestrial animal taxa including invertebrate non-arthropod, arthropod, and vertebrate groups. Species were classified according to their dietary habits following a dietary specialization and mobility gradients. Our objective was to compare responses to fire among taxonomic groups (i.e. species composition per taxon) and functional traits (i.e. dietary-specialization and mobility). We tested whether taxa with a higher proportion of dietary generalists (i.e. omnivorous) had lower responses to fire, that is, whether species composition and abundance were more similar between burnt and unburnt sites (Hypothesis 1). In a broad sense, we assume that omnivores are the most generalist species as they can potentially exploit a wider number of food resources. For this reason, we expect that taxonomic groups having a higher proportion of omnivorous species would be more prone to have responses unrelated to shifts in plant composition (Hypothesis 2). We also tested whether mobility, a key factor in the dispersal capacity of animals, might influence species composition and abundance among groups (Hypothesis 3). Although post-fire recovery may not depend on recolonization rates (see [32]), this attribute is expected to enable species to select adequate habitats in a contrasting (burnt-unburnt) environment.

This study was conducted in a natural park located in north-eastern Spain and affected by a large fire in 2003. Partial results in the park have previously been reported for snails [9], [33]–[34], reptiles [19], and Hymenoptera [35], showing contrasting responses among species to taxonomic and functional levels. These preliminary results encouraged a comparative analysis with the general aim of analysing how responses of a set of animal organisms diverge after being affected by the same disturbance.

This comparative study is meant to identify which groups are more sensitive to postfire habitat changes, and to categorize the sign and magnitude by which each group is affected by fire. The identification of the communities most sensitive to fire is critical to anticipate the impact of projected future changes in the fire regime driven by climate change [36] and thereby guide postfire management efforts to conserve biodiversity (e.g. [37]–[38]).

## Materials and Methods

### Study Area and Fire History

The field work was conducted in Sant Llorenç del Munt i l’Obac Natural Park (Barcelona province, NE Spain). This reserve, located in the Catalan Pre-coastal Mountain Range, has a total area of 13,694 hectares. The park is composed of a polymictic conglomerate ground made up of a deposition of pebbles from a varied origin cemented by an argillaceous and calcareous matrix. The climate of the study area is subhumid Mediterranean with mean annual temperature 12.2°C, ranging from 3.7°C in the coldest month and 22.1°C in the hottest month. Rainfall, reaching around 600 mm annually, is higher in spring and autumn than in summer. Thus, the area is prone to fast-spreading fires during hot, dry summers. The typical forest tree in the Park is Holm oak *Quercus ilex*. However, in peripheral lowland areas of the park, Holm oak was partially replaced by vineyards at the beginning of the 20^th^ century, but, after the devastating *Phylloxera* plague, the fields were abandoned and replaced by Aleppo Pine *Pinus halepensis* and Black Pine *Pinus nigra*. The understory of these pine forests is composed mainly of Holm oak and Mediterranean shrub species.

The study area burned in August 2003 during a summer crown fire that affected 4,443 ha on the eastern border of the park, with 1,778 ha of this lying inside the park. Driven by wind, the entire area burned in just one day (10 August 2003). The burnt area was dominated by pines with small patches of Holm oak forests, abandoned agricultural lands, and scrublands (see more details in [34]). Timber removal began soon after the fire, and two years later most of the area was almost completely logged with only woody debris remaining on the ground. After the logging, a sub-area was also subsoiled to plant mainly coniferous stands.

### Site Selection

**Figure 1:**
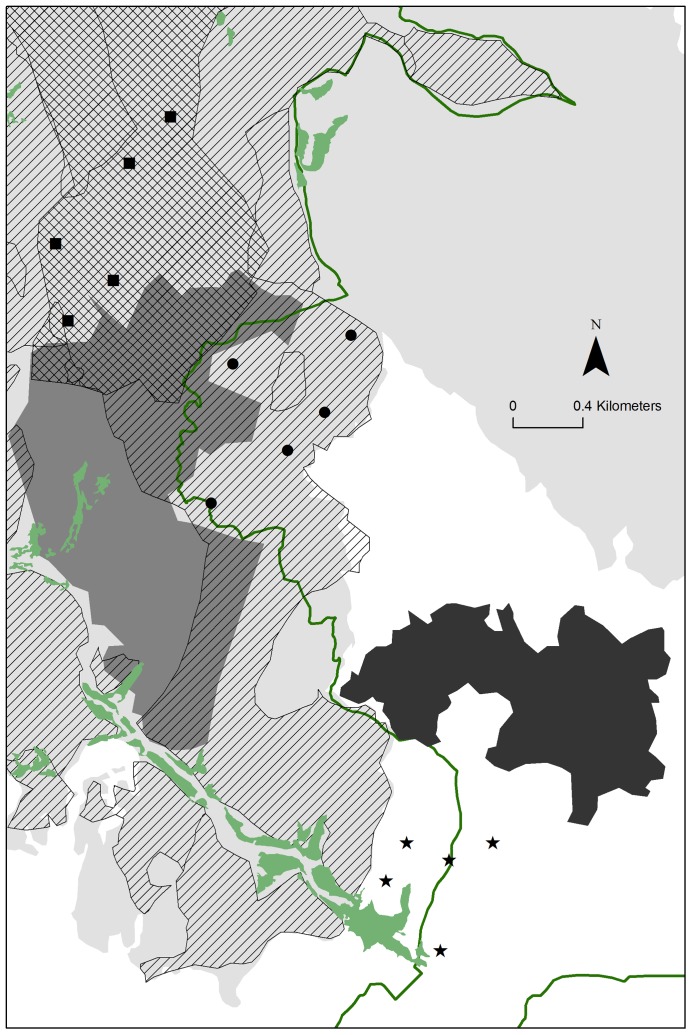
Location of the study area and sampling points. Geographic location of the study area in south-western Europe (A), area burnt in 2003 (grey lines) with respect the Natural Park perimeter (dotted line) (B) and distribution of sites sampled (C). Symbols of sampling points: unburnt area (stars), subsoiling (squares), and logging (circles). Black area: perimeter of Sant Llorenç Savall village; light grey area: perimeter of the fire in 2003; dark grey: perimeter of a previous fire occurred in 1972; green: open areas. Green line: limits of the Natural Park. Postfire management after the fire in 2003: logged (dashed area), and subsoiled (squared area).

Sampling points were selected taking into account postfire management applied in the study area (Figure 1). According to these practices, we defined two different burnt areas: “Logging” was the area burnt only in 2003 with subsequent logging; and “Subsoiling” was the area burnt only in 2003 with subsequent logging and subsoiling. Logging (removal of the burnt tree trunks) and subsoiling (breaking up the soil 60 cm in depth to increase soil volume) may have different impacts on ecosystem function and structure, as well as on animal and plant diversity (see [39] for the logging impact); for this reason, both areas were separately considered in further analyses. Additionally, we established an unburnt reference area (“Unburnt”) in a pine forest near the fire edge with the same dominant tree species of the burnt area before the fire (Figure 1).

Surveys were conducted in five spatial replicates (hereafter, sampling points) per area (Figure 1). Sampling points at the unburnt area and those with the same post-fire practice were spatially clustered since few areas achieved the criteria to be sampled. The five unburnt sampling points were clustered in one area; therefore, the unburnt data would provide an *a priori* weak reference point from which to infer differences between burnt and unburnt sites. However, three main arguments give credibility to the selection of unburnt points: 1) burnt and unburnt sampling points were selected within similar environmental conditions (i.e. orientation, slope, and soil composition), in order to control biases in the sampling design related to factors that might influence fauna and flora composition; 2) three independent criteria (number of trees, snail assemblages, and bird censuses) indicate that environmental conditions and fauna composition before the fire were similar between the unburnt and burnt areas (see more details in the next section); and 3) the distance between the five unburnt points (mean score = 466 m, range 247–689) is far enough for low-mobility species. Although the clustering might affect especially high-mobility species such as birds, Herrando et al. [5] demonstrated in an area less than 100 km far from the Sant Llorenç del Munt Natural Park that the bird species composition and abundance of different unburnt points surrounding a 5000-ha fire were very similar, in contrast to the diverse bird communities within the burnt area.

Furthermore, the main objective of our study was to examine differences among taxonomic and functional groups in a scenario disturbed by the fire, not differences among areas. For this reason, the potential effects of the clustering selection of replicates constrained by the management plans did not compromise the results or further discussion.

### Pre-fire Vegetation Structure

We examined the pre-burnt vegetation structure using an aerial photograph taken in 2001, two years before the fire. At each sampling point, we counted the number of trees in a 50-m buffer and checked differences among sites. This analysis demonstrated that the average number of pines within each buffer did not differ between the three areas (Logging area: mean = 94.6 pines; s.e. 16.4, range 62–151; Subsoiling area: mean = 66.6 pines; s.e. 20.7, range 19–132; Unburnt area: mean = 93.6 pines; s.e. 22.2, range 44–147; Kruskal-Wallis test H = 1.58, d.f. = 2, p = 0.45). Consequently the three areas were considered similar before the 2003 fire in terms of the main element of the habitat structure. Avifaunal surveys made before the 2003 fire (unpublished data) as well as post-fire analysis of shells from dead gastropods collected at the burnt area [9] also suggested that the three areas were similar before the 2003 fire in terms of faunal composition.

### Fauna and Flora Sampling

At each sampling point, we recorded the relative abundance of eight taxonomic animal groups namely Gastropoda (GAS), Heteroptera (HET), Formicidae (FOR), Coleoptera (COL), Araneae (ARA), Orthoptera (ORT), Reptilia (REP), and Aves (BIR). All the animal groups were sampled at the same points during June and July 2007, i.e. four years after the fire. This short sampling period precluded a complete identification of the animal communities (this was not an objective of this study) although it allowed a direct comparison among taxonomic groups. Censuses were taken using the most cost-effective species-specific methods for each taxon (Text S1 in File S1). Vegetation was sampled during the same period, in 20×5 m^2^ field plots in each area and replicate. All the plant species detected (grass, shrubs, and trees) were identified to the species level for further analyses.

The sampling was conducted under permits of the Servei de Biodiversitat i Protecció dels Animals (Direcció General del Medi Natural i Biodiversitat, Catalan Government, Spain) and Sant Llorenç del Munt i l’Obac Natural Park (Diputació de Barcelona, Spain).

### Functional-trait Classification

Species found in the study area were classified according to diet and mobility by researchers who are specialized in each taxonomic group and are authors of this multi-taxonomic study. Diet is only one component of the functional complexity of organisms, although it is key to examine how organisms respond to land-use changes from a functional perspective [40], and it is the easiest trait known for a set of species such as those examined in the present study. Although dietary details are not complete for many species found in the study area, we grouped them according to their dietary habits as zoophagous, phytophagous, saprovorous, or omnivorous. As omnivorous species are predatory and phytophagous at the same time, these species were considered more generalist in their dietary habitats than the others. Taxonomic groups were classified as high-mobility (Aves, Orthoptera and vegetation Coleoptera), medium-mobility (soil and vegetation Formicidae and Araneida), or low-mobility (soil Gastropoda, Reptiles, soil Heteroptera, and soil Coleoptera ) groups. Arthropods were collected with two sampling methods (pitfalls and nets). Due to logistic limitations, only Coleoptera and Formicidae were classified to the species level from specimens collected with both sampling methods as these taxa have high species richness and functional diversity. Pitfalls and net sweeping encompass a very different fraction of arthropod communities (from ground and vegetation, respectively). Therefore, we analyzed separately ant and beetle communities collected by both methods. For the rest of the groups, we classified just the fraction that *a priori* was of most interest in terms of functional diversity, species richness or singularity.

### Statistical Procedures

For each sampling point and animal group, we measured the total number of specimens, species richness, and evenness. Evenness refers to how close in numbers each taxonomic group in a sampling point is, and was measured as the reciprocal form of Simpson’s index divided by the number of species in the sample [41]. Evenness ranged from 0 to 1 (the lower the variation in communities between the species, the higher the evenness). For each animal group, species richness and evenness per sampling point were compared among the three areas by an ANOVA or the Kruskal-Wallis test after checking the homogeneity of variances by the Levene test. Overall species richness and evenness per sampling point were also calculated pooling the data of all the animal groups. When differences were significant, *post hoc* comparisons were checked with Student-Newman-Keuls tests.

Relative abundance data recorded per sampling point were then analysed at three different levels:

1. At a taxonomic level, animal species were grouped into 10 taxonomic categories (soil and vegetation Coleoptera and Formicidae were considered separately). Given the low number of replicates per area, we removed from taxonomic analyses (PCA and ANOSIM) those animal species with very low occurrences (less than three records on the complete sampling). A high number of rare species can affect similarity analyses between pairs of sites. With this procedure, we avoided biases by excluding species with very low occurrence due to their scarcity or low detectability.

From the animal species abundance matrix at each sampling point, we carried out a principal component analysis (PCA) to show the similarity in animal composition between areas. Animal species scores were log-transformed (log x+1) and divided by the standard deviation (see [42]) in order to avoid differences in species abundance due to different sampling methodologies used with soil animals (pit-fall traps) and vegetation animals (sweep-netting). The PCA analysis was made using CANOCO software [42].

Differences in animal-species composition (using abundance data) between pairs of sampling points were quantified by the Bray-Curtis similarity index. From the similarity matrix, we performed an ANOSIM [43], which gives a general R-value and allows pairwise comparisons between the three areas. In an effort to avoid biases due to the existence of aggregate species with large sample sizes at a single sampling point, before the similarity and ANOSIM analyses, relative abundances of soil and vegetation Coleoptera and Gastropoda were sqr-transformed, and relative abundance values of soil and vegetation Formicidae and total fauna were log-transformed (log x+1).

To compare responses to fire among animal communities, we used the scores of the R statistics found in the pairwise comparisons of the ANOSIM in a principal component analysis (PCA); those communities with similar responses were expected to show similar R scores in the pairwise comparisons between areas and then to be grouped in the biplot of the two more explicative factors of the PCA.

1. At a functional level, and within each taxonomic group, the matrix of dietary-trait abundance values was examined following the same procedures as for the species-abundance matrix: firstly, we performed a PCA with abundance values of each feeding group per sampling point; secondly, we quantified differences between areas by the Bray-Curtis similarity index, and thirdly, we performed an ANOSIM.
2. Finally, we compared the taxonomic and functional responses between animal groups and vegetation. This relationship was analysed using a battery of Mantel tests. To perform this analysis, we first created a vegetation-similarity matrix comparing plant composition between pairs of the 20×5 m^2^ field plots. The Mantel test then compared the Bray-Curtis similarity matrix of vegetation with the Bray-Curtis similarity matrix of each faunal group (abundance); each comparison gave a Rho statistic and p values which summarized differences between matrices. These element-by-element correlations of two similarity matrices were conducted by the RELATE routine in PRIMER software [43].

To check our hypotheses, the general R-value (taxonomic similarity among areas) and the Rho statistic (similarity in the responses between plants and animals) calculated for each animal group were correlated to the total percentage of omnivorous species found within each group (Hypothesis 1 and 2). We also checked whether similarities among areas for each taxonomic group were related to mobility patterns (Hypothesis 3).

## Results

### Taxonomic Comparisons

Overall, we recorded 4606 individuals from 274 animal species (26 molluscs, 213 arthropods and 35 vertebrates, Table S1 in File S1) and identified 135 plant species (Table S2 in File S1). The mean number of animal species recorded per site did not differ among areas (ANOVA test F_2,14_ = 1.505, *P* = 0.26) whereas evenness marginally differed (F_2,14_ = 3.647, *P* = 0.06), being higher in the unburnt area. For each animal group, the comparison in species richness and evenness among the three areas showed different responses: for some groups, the highest scores were at unburnt sites (e.g. Gastropoda and Aves in species richness), whereas for others at burnt sites (e.g. vegetation Coleoptera). Araneae had higher species richness in the area subsoiled, and higher evenness in the area logged. We found no differences for the rest of the groups (Figure S1 in File S1).

The total number of species (and specimens) found was 147 (1836) at Logging, 140 (1613) at Subsoiling, and 161 (1157) at Unburnt areas. After removing animal species with a low number of individuals, we maintained for further analyses 4439 individuals from 152 species (105 in area Logging, 102 in area Subsoiling, and 103 in area Unburnt). The number of species found exclusively in one area was low: 5 species in Logging, 10 in Subsoiling, and 24 in Unburnt, this result indicating that most species were likely found in at least two different areas. The ANOSIM analysis showed that all the animal assemblages except vegetation Formicidae, Orthoptera, and soil Coleoptera differed overall among sampling areas (R statistic and percentage of the adjusted statistic <5%, Table S3 in File S1). When all the animals for each sampling point were pooled, pairwise comparisons also proved significant, hence demonstrating that the faunal composition and species abundance differed among the three areas. ANOSIM scores were similar for the overall and taxonomic groups when rare species were not excluded. Vegetation showed significant differences in plant composition except for the comparison between the areas Logging and Subsoiling (Table S3 in File S1).

**Figure 2:**
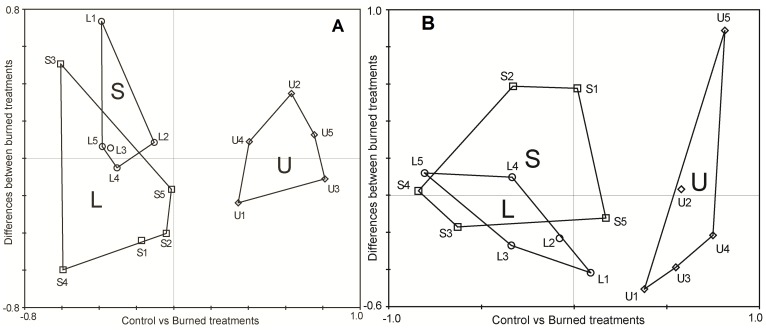
Principal component analysis biplots according the taxonomic and functional responses to fire. (A) PCA based on the standardized relative abundances of 152 animal species sampled after removing rare species (less than three records) at the 15 sampling points (numbered from 11 to 35). Each number indicates the position of one sampling site, and the polygons join sites of the same area. Points U1 to U5 = unburnt pine forest (“U”), L1 to L5 = logging (“L”), S1 to S5 = subsoiling (“S”). Axis 1 (horizontal) = 21.2% of explained variance, axis 2 (vertical) = 11.8%. (B) PCA based on the abundances of the four feeding groups (zoophagous, phytophagous, saprovorous, and omnivorous) collected at the 15 sampling points (numbered from 11 to 35). Each number indicates the position of one sampling site, and the polygons join sites of the same area. Points U1 to U5 = unburnt pine forest (“U”), L1 to L5 = logging extraction (“L”), S1 to S5 = subsoiling (“S”). Axis 1 (horizontal) = 27.5% of explained variance, axis 2 (vertical) = 15.3%.

**Figure 3:**
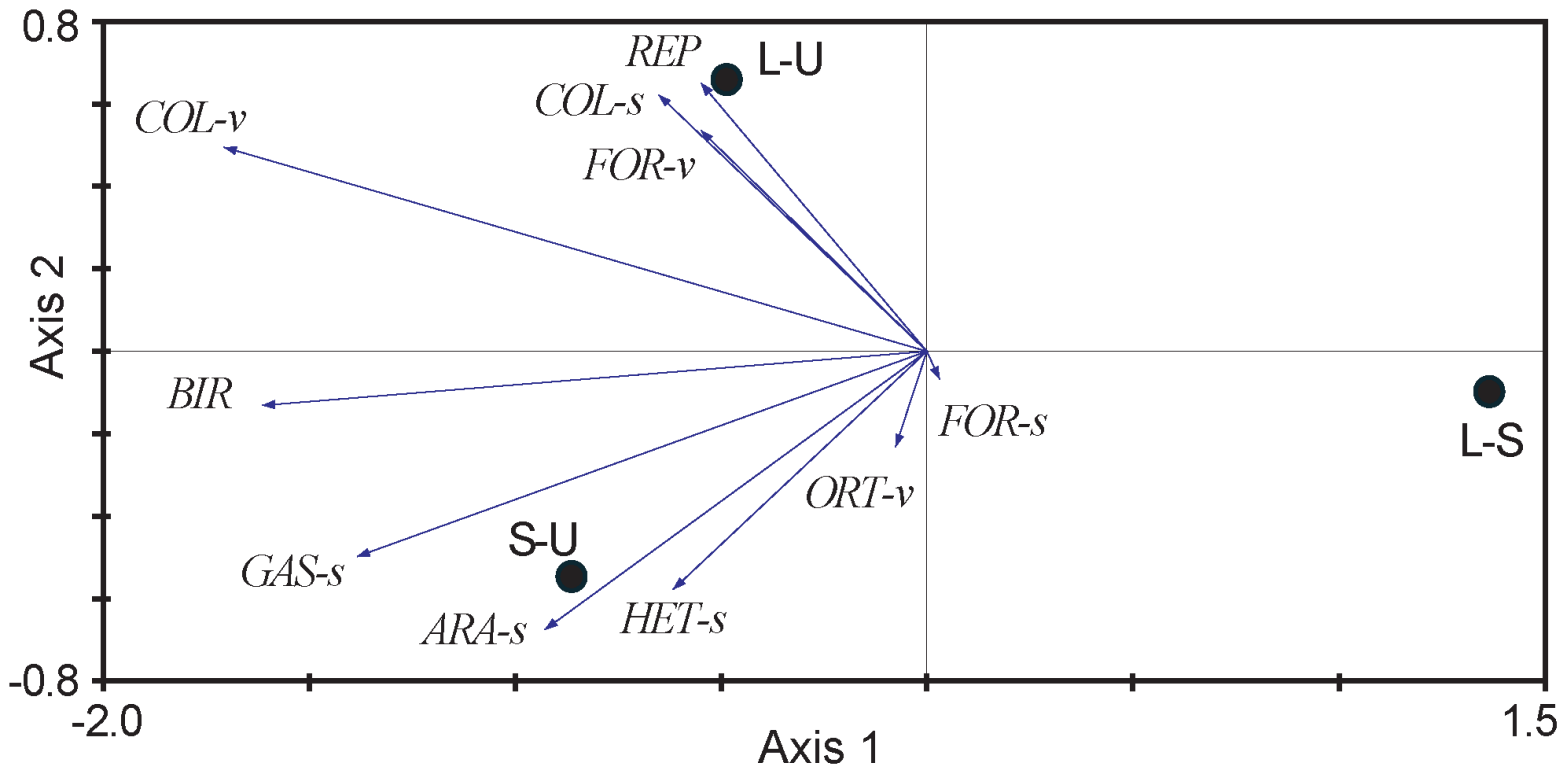
Principal Component Analysis biplot based on R scores of the pairwise comparisons between the three areas for each animal group. Axis 1 (horizontal) = 94.0% of variance explained, axis 2 (vertical) = 6.0%. Acronyms of the three areas: unburnt “U”, logging “L”, and subsoiling “S”. Acronyms of animal groups: Gastropoda (GAS), Heteroptera (HET), Formicidae (FOR), Coleoptera (COL), Araneida (ARA), Orthoptera (ORT), Reptilia (REP), and Aves (BIR). Animal group acronym followed by –v or –s means vegetation or soil group respectively. Except FOR-s and ORT-v, animal groups tend to have their maximum R scores in burnt (L or S) versus unburnt (U) areas in the ANOSIM pairwise comparisons.

In the PCA biplot drawn from the relative abundance values of the 152 animal species, axis 1 (variance explained = 21.2%) clearly discriminated the unburnt area from the two burnt areas. The two burnt areas had more similar species composition (Figure 2A). In the PCA biplot drawn with the R scores of the pairwise comparisons between the three areas, the first axis explained a significant amount of variation (variance explained = 94.0%) and discriminate between unburnt and burnt areas (Figure 3). Animal groups showed a gradient of taxonomic response to fire: Aves, vegetation Coleoptera and Gastropoda were the groups with the strongest response to fire (positive values in axis 1, Figure 3), and soil Formicidae and Orthoptera showing the lowest response.

### Dietary Functional Comparisons

**Figure 4:**
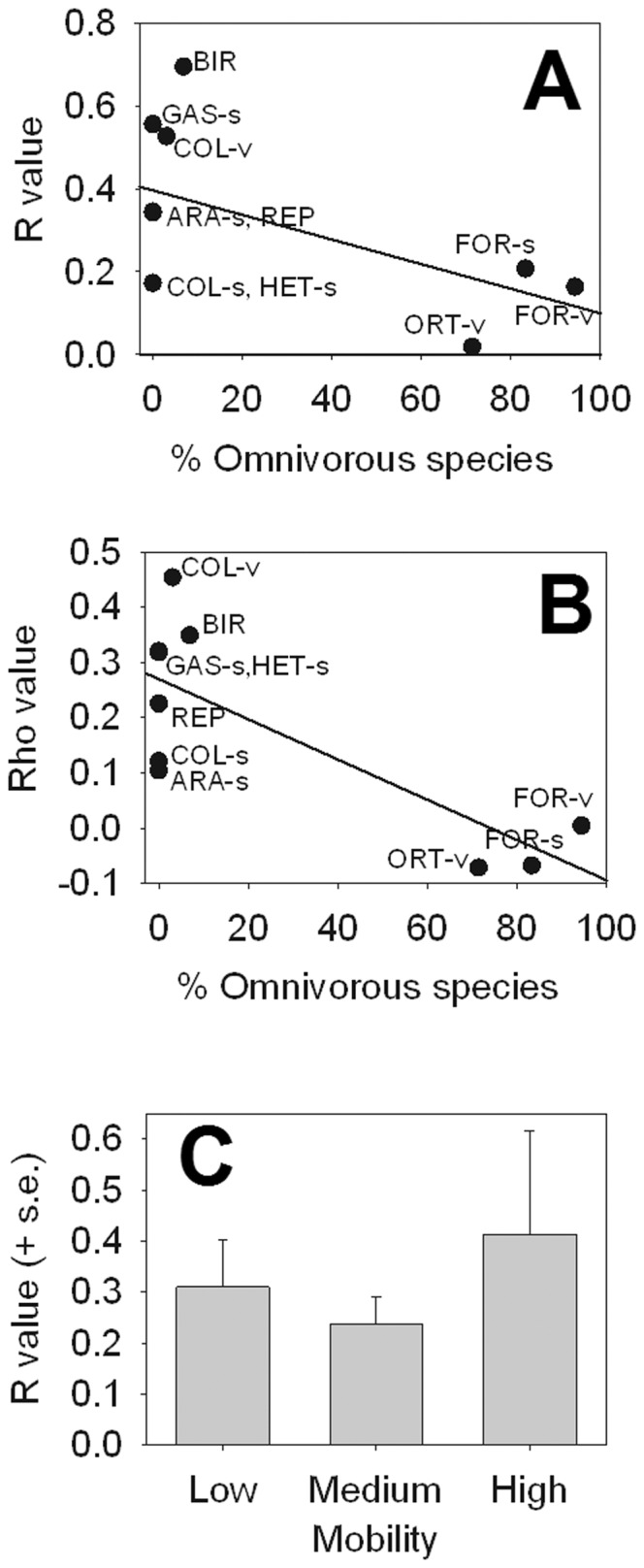
Figures of the three hypotheses tested. (A) Correlation between the proportion of omnivorous species within each taxonomic group and their taxonomic differences, i.e. R scores, among areas (Hypothesis 1). R values were calculated by ANOSIM; higher scores mean higher differences in species abundance and composition among areas. Pearson correlation line is showed. (B) Correlation between the proportion of omnivorous species within each group and their plant-animal similarity responses, i. e. Rho scores, among areas (Hypothesis 2). Rho values were calculated by Mantel tests; higher scores means more similar responses between plants and animals. Pearson correlation line is showed. (C) Differences in R values for the taxonomic groups classified according to their mobility (Hypothesis 3). R values were calculated with ANOSIM; higher scores mean higher differences in species abundance and composition among areas.

At a functional level, the highest number of species corresponded to zoophagous and phytophagous dietary types, whereas the abundance of phytophagous and omnivorous individuals was larger (Table 1). The PCA biplot drawn from the relative abundances of the four functional dietary classes per sampling point, clearly discriminated the unburnt area from the two burnt areas (axis 1; variance explained = 27.5%), the two burnt areas having more similar dietary composition (Figure 2B; Table 1). The ANOSIM showed differences in the abundance of the four dietary types between unburnt and the two burnt areas (Table S4 in File S1), especially due to the highest proportion of zoophagous animals in the unburnt area (Table 1). For each animal group, only Araneae, Aves, and vegetation Coleoptera showed significant variation in the overall proportion of functional groups between areas (R statistic and percentage of the adjusted statistic <5%, Table S4 in File S1). We found no significant correlation between the proportion of omnivorous species within each taxonomic group and their taxonomic and functional differences among areas, i.e. R scores (Hypothesis 1; Figure 4A; Spearman correlation, *P*>0.5).

**Table 1:** Number of species and specimens according to diet.

Number of species and percentage per area				
	“U”	“L”	“S”	Total
Zoo	51 (31.7)	40 (27.2)	46 (32.9)	90 (32.8)
Phy	54 (33.5)	61 (41.5)	58 (41.4)	105 (38.3)
Sap	17 (10.6)	8 (5.4)	9 (6.4)	23 (8.4)
Omn	39 (24.2)	38 (25.9)	27 (19.3)	56 (20.4)
Total	161	147	140	274
**Number of specimens and percentage per area**				
	**“U”**	**“L”**	**“S”**	**Total**
Zoo	175 (15.1)	115 (6.3)	136 (8.4)	426 (9.2)
Phy	418 (36.1)	528 (28.8)	450 (27.9)	1396 (30.3)
Sap	32 (2.8)	70 (3.8)	85 (5.3)	187 (4.1)
Omn	532 (46.0)	1123 (61.2)	942 (58.4)	2597 (56.4)
Total	1157	1836	1613	4606

Zoophagous (Zoo), phytophagous (Phy), saprovorous (Sap), and omnivorous (Omn). For each area, the five sampling points were pooled. Abbreviations of the three areas are unburnt (“U”), logging (“L”), and subsoiling (“S”).

### Comparison of Animal and Plant Composition

The similarity matrix between sampling points based on presence/absence of plant species and similarity matrices based on abundances of each animal group, were compared by Mantel tests. The Rho statistic of each comparison showed a significant association between vegetation and fauna in four out of 10 groups, namely Aves, Gastropoda, vegetation Coleoptera and soil Heteroptera (Table 2). Notably, taxonomic groups with higher Rho values (more similar responses between animals and plants) had a lower number of omnivorous (generalist) species (Pearson correlation r = −0.768, *P* = 0.009; Figure 4B) and specimens (r = −0.750 *P* = 0.01). This result suggests that the response of animals to fire is likely governed by changes in vegetation composition for those groups with a higher number of dietary specialists (Hypothesis 2).

**Table 2:** Mantel test results (Rho statistic) of the comparison between similarity matrices of vegetation and taxonomic faunal groups.

			Species				Specimens			
	Rho	P	%Zoo	%Phy	%Sap	%Omn	%Zoo	%Phy	%Sap	%Omn
GAS SOIL	0.320	0.0022	7.7	76.9	15.4	0.0	1.9	95.7	2.5	0.0
ARA SOIL	0.104	0.2510	100.0	0.0	0.0	0.0	100.0	0.0	0.0	0.0
HET SOIL	0.318	0.0009	25	75	0.0	0.0	34.1	65.9	0.0	0.0
FOR SOIL	−0.068	0.6652	6.7	10	0.0	83.3	0.8	6.3	0.0	92.9
FOR VEG	0.004	0.4873	0.0	5.6	0.0	94.4	0.0	57.6	0.0	42.4
COL SOIL	0.121	0.1974	22.2	29.6	48.1	0.0	7.3	16.7	76.0	0.0
COL VEG	0.454	0.0004	17.2	70.3	9.4	3.1	7.1	81.2	5.4	6.3
ORT VEG	−0.072	0.6857	0.0	28.6	0.0	71.4	0.0	37.4	0.0	62.6
REP	0.225	0.0992	100.0	0.0	0.0	0.0	100.0	0.0	0.0	0.0
BIR	0.349	0.0080	62.1	31	0.0	6.9	67.8	29.9	0.0	2.3
Total Fauna	0.299	0.0099	32.8	38.3	8.4	20.4	9.2	30.3	4.1	56.4

Similarity matrices were constructed with the Bray-Curtis index. Rho = Rho statistics of RELATE routine, p = Monte Carlo Permutation test of significance (9999 permutations). %Zoo = percentage of zoophagous species/specimens, %Phy = percentage of phytophagous species/specimens, %Sap = percentage of saprovorous species/specimens, %Omn = percentage of omnivorous species/specimens. Phytophagous category include species that feed on mushrooms or any plant part (i.e. leaves, wood, roots, seeds, pollen, and nectar). For abbreviations of groups, see text (Material and methods).

### Mobility Functional Comparisons

The highest mobility and lowest mobility groups (Aves and Gastropoda, respectively) showed the strongest response to fire (positive values in axis 1, Figure 3 **)**. We found a pattern accounting for higher responses to fire (higher differences) in high- and low-mobility than in medium-mobility groups (Hypothesis 3; Figure 4C); these differences, however, had no statistical support (Kruskal-Wallis test H = 0.704, d.f. = 2, *P* = 0.76).

## Discussion

Our study, made in an early stage of the post-fire succession, highlights notable differences in animal composition between burnt and unburnt areas. Although the sampling design was constrained by the clustered distribution of replicates, especially the unburnt ones, there are several independent pieces of evidence showing that sites were similar in fauna and flora prior to the fire [5]–[9]; this finding gives more confidence that our results represent a real effect of past burning. The sampling design precludes to conclude that differences between burnt and unburnt areas are exclusively the result of fire. However, similarity among areas in pre-fire habitat and fauna, and differences in post-fire habitat structure supports the contention that variation in animal composition is related to fire. The low number of exclusive species suggests that differences are due primarily to the replacement of dominant species among areas. It is known that postfire simplification in vegetation structure may cause replacements of dominant species [5]–[9], which, as the present study shows, can affect a diverse array of animal assemblages.

Comparing responses to fire among the animal groups, we found clear evidence that animal communities did not respond uniformly to early postfire succession, as reported by [44] in arthropod communities in temperate forests. We found a response gradient, with some taxonomic groups showing a strong response to fire (Aves, vegetation Coleoptera, and Gastropoda) whereas other groups did not (Orthoptera, Formicidae). The fact that different groups responded differentially to the same disturbance emphasizes the importance of taking into account the degree to which a taxon may be used as a surrogate for the effects of disturbances on the whole ecosystem. Importantly, this specific-taxon variation seems at least partially related to functional traits of each group.

### Responses to Fire According to the Number of Generalist Species

The Mantel tests suggest that plants and animals showed similar trends in their responses to fire (see also [45]). This conclusion supports the general acceptance that habitat attributes (vegetation structure and cover) may be keystones of animal responses to fire (e.g. [45]–[46]). We failed to detect a correlation between the response to fire (overall R values for each animal group) and proportion of generalist species (Hypothesis 1). However, we found a significant correlation in the similarity of animal-plant responses and the proportion of generalist species (Hypothesis 2) as groups with large numbers of omnivorous (generalist) species (i.e. Formicidae and Orthoptera, Table 2) had more vegetation-independent responses. For omnivorous species, postfire shifts in plant composition appears to be unimportant, compared to feeder-specialist species. As expected, Formicidae and Orthoptera were also the most resilient groups, this indicating a link between resilience and non-specialization in functional traits. Resilience, the ability of a species/community/system to recover from an environmental change [47], has been linked to the diversity of responses [48] at the ecosystem level, to diversity and heterogeneity [49] at the community level, and to particular functional traits (e.g. generalist species) [50] at the species level. Ecological theory predicts that specialist species would be favoured in stable systems whereas abrupt environmental changes triggered by disturbances would promote generalist species [29]. Based on a set of species and taxonomic groups, our results indicate that fire favoured the maintenance of generalist (omnivorous) species, this fact reflecting high resilience in these taxa. However, strong taxonomic differences in the majority of groups suggest a replacement of non-generalist species from unburnt to burnt habitats. This apparent contradiction of the theory has been detected in a bird community [51], the authors arguing that ecological specialization cannot be measured in a single gradient due to the multidimensionality of the ecological niche, and particular characteristics of each type of disturbance may have specific consequences on the responses of communities. This high degree of heterogeneity make it difficult to build general ecological trends based on the response of organisms to disturbance, and underscores the value of comparing the response of a number of taxonomic groups to a single disturbance, as shown in the present study.

### Response to Fire According to Animal Mobility

Within the taxonomic groups examined, Aves was the group with the highest mobility and the strongest response to fire at taxonomic and functional levels. Its high species and functional diversity, and sensitivity to habitat structure make birds a valuable indicator of habitat changes [52]. As expected, Aves react markedly to environmental changes due to their recolonization abilities in burnt and postfire managed burnt habitats [7]–[20]. However, other groups with lower mobility such as Gastropoda and Araneida also proved to be good indicators of habitat variation in fire-prone areas (see also [53], [54]–[55]). Unexpectedly, Gastropoda and Aves showed a similar response with lower species richness in burnt than unburnt sites. For Gastropoda, this pattern may be caused by the high mortality directly inflicted by fire and low recolonization rates [9] despite the availability of cryptic refuges in burnt areas [56]. For Aves, this pattern appears to be related to the higher complexity of the habitat structure at unburnt sites coupled with their dispersal capacity along the postfire succession [7]–[57]. The similar response displayed by taxonomic groups with contrasting activity and dispersal patterns (i.e. Aves and Gastropoda) illustrates how fire exerts strong effects from the microhabitat to the landscape scale. We found no statistical support for differences among groups according to their mobility (Figure 4C), probably due to a high variability of this functional trait among species of a particular taxon.

### Biodiversity Conservation and Habitat Heterogeneity

Species replacement in our study area suggests expansion into burnt areas of threatened species such as the gastropod *Xerocrassa montserratensis* [58], and the bird *Alectoris rufa* (red-legged partridge) [59]. These results support the idea that fire may play a critical role for some threatened Mediterranean species [60], [24]–[33]. In the Mediterranean basin, during the second half of the 20^th^ century the coniferous forest area increased by pine forestation as well as by natural pine recolonization due mainly to land abandonment [3]. In Catalonia (NE Spain) this increase reached 130% [61]. Most wildfires in the Mediterranean basin are currently affecting these pine forests. The short-term direct consequence is the recovery of open areas and, as our results show, the expansion and recolonization of open-habitat species of a wide variety of taxa. This is particularly important, since European biodiversity indicators for species of vertebrates (e.g. birds) and invertebrates (e.g. butterflies) have shown sharp declines of open-specialist species in the last few decades [62]–[63]. This trend suggests that early postfire-succession stages coupled with other processes such as traditional land uses, may play a role in the overall conservation of biodiversity in the Mediterranean basin. The current trend of the abandonment of traditional agriculture in this region [64] can magnify the usefulness of manipulative experiments such as prescribed fires to manage the conservation of Mediterranean biodiversity, as has been reported in other regions of the world [65].

Although fire promotes the expansion of some threatened open-area species, many species exclusive of unburnt sites in the study area were negatively affected by fire. In fact, fire fosters landscape heterogeneity [49], creating a mosaic of open, forest, and ecotonal areas. The positive role of habitat heterogeneity on biodiversity is a well-known and predictable rule in ecology [66]–[67]. To maximize biodiversity, habitat management may provide mosaics of open and forest areas [68], hence maximizing the expansion of both open- and forest-specialist species and thus improving the changes of colonization of burnt areas after new disturbances [7].

### Conclusions and Perspectives for Conservational Management Plans

Our study has demonstrated that, four years after the fire, the majority of animal groups and plants surveyed reflect significant differences between burnt and unburnt sites, and also several differences among burnt areas submitted to different postfire management practices. Differences are driven primarily by vegetation, as we found complementary patterns in plant and animal composition, especially for those animal groups with many specialist species in feeding habits. The increased diversity at a landscape level and the link between vegetation and animal patterns in disturbed areas give extraordinary importance to postfire management practices (see for example [69]). The main objective of managers in wildlife reserves is to maintain the natural biodiversity, emphasizing the preservation of endemic species. Our study as well as other previous works demonstrate that the maintenance of landscape heterogeneity of fire mosaics may be an appropriate management practice [70], [37]. In areas historically disturbed by humans and severely affected by fire, future experimental management and subsequent wildlife monitoring should be conducted in order to 1) validate the effectiveness of practices in terms of benefits for biodiversity, 2) recognize ecological mechanisms related to animal-plant interactions in successional trajectories, and 3) identify reliable indicators (e.g. species interactions and particular species) of postfire processes.

## Supporting Information

File S1 **Supporting information file including Text S1, Tables S1–S4, and Figure S1. Text S1. Description of the sampling methods. Table S1**. **Basic data of the 15 sampled plots and abundances of animal species**: burnt logging (L1 to L5), burnt subsoiling (S1 to S5) and unburnt (U1 to U5); Data: species included (Y) and excluded (N) in statistical analyses according to the number of records. FG (feeding groups): zoophagous (1), phytophagous (2), saprovorous (3) and omnivorous (4). **Table S2**. **List of plant species and presence in the three areas**: logging (“L”), subsoiling (“S”) and unburnt (“U”). Table S3. R values and significance (* denotes p<0.05) from the ANOSIM taxonomic analysis of each animal group. The last rows are R values for the overall animal (abundance) and plant (presence/absence) species. The Global R column indicates the overall comparison of the three areas. The rest of the columns indicate the pairwise comparison between areas, with the R value and significance. Acronyms of the three areas are unburnt reference (“U”), logging (“L”) and subsoiling (“S”). For acronyms of groups, see text. **Table S4.** R values and significance (* denotes p<0.05) from the ANOSIM functional (dietary) analysis of each animal group. The Global R column indicates the overall comparison among the three areas. The rest of the columns indicate the pairwise comparison between areas, with the R value and signification. Acronyms of the three areas are unburnt reference (“U”), logging (“L”) and subsoiling (“S”). For acronyms of groups, see text. **Figure S1.** **Comparison of the total number of animal species and evenness among areas for each animal group:** unburnt “U”, logging “L” and subsoiling “S”. Each column represents average scores ± standard error. Each figure includes the ANOVA or Kruskall-Wallis tests, and letters refer to post hoc comparisons between areas.
(DOCX)
