# Supplementary material for: Is Response to Fire Influenced by Dietary Specialization and Mobility? A Comparative Study with Multiple Animal Assemblages
Source: PLoS One. 2014 Feb 7;9(2):e88224. doi: 10.1371/journal.pone.0088224 (PMC3917858; doi:10.1371/journal.pone.0088224)

**Supporting Information File S1**

**Text S1. Description of the sampling methods.**

Sampling methods: Gastropoda were searched for at each sampling point by two complementary methods (Santos et al. 2009): 1) all snails over 5 mm were searched for actively 30 min in a 10x10 m area in the centre of the site; 2) snails smaller than 5 mm were collected from one 25x25 cm subsample of litter and in the top 5 cm of soil profile. The subsample was randomly located within the large 10 × 10 m square, and examined in the laboratory with a microscope to count and identify small snails.

Soil arthropods (Araneae, Coleoptera, Formicidae and Heteroptera) were collected using pitfall traps, a standard and efficient method for collecting ground-dwelling arthropods (Drake et al. 2007). Five traps were installed at each sampling point, placed at 10-m intervals in a straight line. After 15 days the traps were removed and the arthropods were preserved in a 70% alcohol. The traps used consisted of a plastic collector of 7.5 cm in diameter and 10 cm in depth, placed within a plastic container of 10 cm in diameter and 15 cm deep. A plastic funnel of 8 cm in diameter was inserted over the collector pot. A supersaturated aqueous salt solution was used as a preservative. Pitfall-trap captures depend on both animal activity and density, and therefore the results reflect a mixture of the above two parameters (e.g. Lensing et al. 2005; De Mas et al. 2009).

Vegetation arthropods (Coleoptera, Formicidae and Orthoptera) were collected by sweep netting. We gathered five random samples from each sampling point. Each sample consisted of sweeping (20 sweeps) while walking at a constant speed along a straight path. The arthropods captured were removed from the net using an entomological aspirator and forceps, and preserved in 70% alcohol. The net sweep used in the present study, 50 cm deep, had a light frame of 40 cm in diameter mounted on a 1-m-long pole. For entomological studies in vegetation, insect nets are fundamental because they capture many diverse groups of arthropods (basically insects and arachnids) in a short time (Drake et al. 2007). Soil and vegetation arthropods were separately classified by taxonomic category of order, and later several groups were identified to the species level.

Reptiles were surveyed at each sampling point by searching for active specimens or those hidden inside vegetation or refuges for 30 min during sunny and hot days. Surveys were made in an area of approximately 200 m diameter around the centre of the sampling site, zigzagging to avoid resightings. These 200-m buffers around each site delimit the total area sampled by active searching.

Birds were sampled at each sampling point using a line-transect method (Bibby et al. 2000). Each transect was sampled in the morning during the maximum activity of this group. Transects were sampled at an average speed of 1-2 km/h, for 15 min, crossing the centre of each site.

Formicidae and Coleoptera from soil and vegetation (FOR-s, FOR-v, COL-s, COL-v) were collected with two different methods (pitfall traps for soil assemblages and sweep netting for vegetation assemblages). We considered the two methods to be different taxonomic categories when we performed the analysis at taxonomic and functional levels (see Statistical procedures).

Despite of the use of different sampling methods applied for each taxon, comparisons between groups are adequate since plant and animal data were collected at the same sites and under the same spatial design of burnt-unburnt and burnt managed areas.

References

Bibby, C. et al. 2000. Bird census techniques. - Cambridge Univ. Press.

De Mas, E. et al. 2009. Resurrecting the differential mortality model of sexual size dimorphism. – J. Evol. Biol. 22: 1739-1749.

Drake, C. M. et al. 2007. Surveying terrestrial and freshwater invertebrates for conservation evaluation. - Natural England Research Report NERR005. Natural England

Lensing, J. R. et al. 2005. The impact of altered precipitation on spatial

stratification and activity-densities of springtails(Collembola) and spiders (Araneae). – Ecol. Entomol. 30: 194-200.

Santos, X. et al. 2009. Recolonization of a burnt Mediterranean area by terrestrial gastropods. – Biodiv. Conserv. 18: 3153-3165.

**Table S1**. **Basic data of the 15 sampled plots and abundances of animal species**: burnt logging (L1 to L5), burnt subsoiling (S1 to S5) and unburnt (U1 to U5); Data: species included (Y) and excluded (N) in statistical analyses according to the number of records. FG (feeding groups): zoophagous (1), phytophagous (2), saprovorous (3) and omnivorous (4).

| **Site** | **Replicate** | **Altitude (m)** | **Slope (%)** | **Orientation** | **UTM X** | **UTM Y** |
| --- | --- | --- | --- | --- | --- | --- |
| **Logging** | L1 | 601 | 35 | S | 419682 | 4616897 |
| **Logging** | L2 | 556 | 27 | E | 420355 | 4617055 |
| **Logging** | L3 | 576 | 30 | SE | 420241 | 4616759 |
| **Logging** | L4 | 617 | 28 | SE | 419998 | 4616413 |
| **Logging** | L5 | 662 | 36 | E-SE | 419521 | 4616051 |
| **Subsoiling** | S1 | 664 | 32 | SE | 418655 | 4617567 |
| **Subsoiling** | S2 | 676 | 42 | E | 418723 | 4617125 |
| **Subsoiling** | S3 | 651 | 32 | S | 419300 | 4618310 |
| **Subsoiling** | S4 | 626 | 29 | S | 419100 | 4617789 |
| **Subsoiling** | S5 | 702 | 29 | E | 418882 | 4617110 |
| **Unburnt** | U1 | 518 | 35 | E-SE | 420595 | 4613902 |
| **Unburnt** | U2 | 519 | 27 | SE | 420760 | 4614153 |
| **Unburnt** | U3 | 481 | 18 | S | 420936 | 4613718 |
| **Unburnt** | U4 | 534 | 19 | SE | 421080 | 4614272 |
| **Unburnt** | U5 | 504 | 21 | E-SE | 420906 | 4614088 |

| **Taxon** | ***Species*** | **L1** | **L2** | **L3** | **L4** | **L5** | **S1** | **S2** | **S3** | **S4** | **S5** | **U1** | **U2** | **U3** | **U4** | **U5** | **Data** | **FG** |
| --- | --- | --- | --- | --- | --- | --- | --- | --- | --- | --- | --- | --- | --- | --- | --- | --- | --- | --- |
| ARA-s | *Alopecosa sp2* | 0 | 0 | 0 | 0 | 0 | 0 | 0 | 0 | 0 | 0 | 0 | 0 | 1 | 0 | 0 | N | 1 |
| ARA-s | *Callilepis sp* | 0 | 0 | 1 | 1 | 0 | 7 | 1 | 0 | 3 | 1 | 0 | 0 | 0 | 0 | 0 | Y | 1 |
| ARA-s | *Euophrys herbigrada* | 0 | 0 | 0 | 1 | 0 | 0 | 0 | 0 | 0 | 0 | 0 | 0 | 0 | 0 | 0 | N | 1 |
| ARA-s | *Gnaphosidae sp1* | 0 | 0 | 0 | 0 | 0 | 0 | 1 | 1 | 0 | 1 | 0 | 0 | 0 | 0 | 0 | Y | 1 |
| ARA-s | *Gnaphosidae sp10* | 0 | 0 | 0 | 0 | 0 | 0 | 0 | 0 | 0 | 0 | 0 | 0 | 1 | 0 | 1 | N | 1 |
| ARA-s | *Gnaphosidae sp11* | 0 | 1 | 0 | 0 | 0 | 0 | 0 | 0 | 0 | 0 | 0 | 0 | 0 | 0 | 0 | N | 1 |
| ARA-s | *Gnaphosidae sp12* | 0 | 0 | 0 | 0 | 0 | 0 | 0 | 0 | 0 | 0 | 0 | 0 | 0 | 1 | 0 | N | 1 |
| ARA-s | *Gnaphosidae sp13* | 0 | 0 | 0 | 0 | 0 | 1 | 0 | 0 | 0 | 0 | 0 | 1 | 0 | 0 | 0 | N | 1 |
| ARA-s | *Gnaphosidae sp14* | 0 | 0 | 0 | 0 | 0 | 0 | 1 | 0 | 0 | 0 | 0 | 0 | 0 | 0 | 0 | N | 1 |
| ARA-s | *Gnaphosidae sp2* | 0 | 0 | 0 | 0 | 2 | 0 | 0 | 2 | 0 | 0 | 0 | 0 | 0 | 0 | 0 | Y | 1 |
| ARA-s | *Gnaphosidae sp4* | 1 | 0 | 0 | 0 | 0 | 0 | 0 | 1 | 2 | 1 | 0 | 0 | 0 | 0 | 0 | Y | 1 |
| ARA-s | *Gnaphosidae sp8* | 0 | 0 | 0 | 0 | 0 | 1 | 0 | 0 | 0 | 0 | 1 | 0 | 0 | 0 | 0 | N | 1 |
| ARA-s | *Gnaphosidae sp9* | 0 | 0 | 0 | 0 | 0 | 0 | 1 | 2 | 1 | 0 | 0 | 0 | 0 | 1 | 0 | Y | 1 |
| ARA-s | *Hahnia sp1* | 0 | 0 | 0 | 0 | 0 | 0 | 0 | 0 | 0 | 0 | 0 | 1 | 0 | 0 | 0 | N | 1 |
| ARA-s | *Haplodrassus umbratilis* | 1 | 0 | 0 | 0 | 0 | 0 | 0 | 0 | 0 | 0 | 0 | 0 | 0 | 0 | 0 | N | 1 |
| ARA-s | *Hogna radiate* | 1 | 0 | 0 | 0 | 0 | 0 | 1 | 0 | 0 | 1 | 0 | 0 | 0 | 2 | 0 | Y | 1 |
| ARA-s | *Linyphiidae sp1* | 0 | 0 | 0 | 0 | 0 | 0 | 0 | 0 | 0 | 0 | 1 | 0 | 0 | 0 | 0 | N | 1 |
| ARA-s | *Linyphiidae sp2* | 0 | 0 | 0 | 0 | 0 | 0 | 0 | 0 | 0 | 0 | 0 | 1 | 0 | 0 | 1 | N | 1 |
| ARA-s | *Linyphiidae sp3* | 0 | 0 | 0 | 1 | 0 | 0 | 0 | 0 | 0 | 0 | 0 | 0 | 0 | 0 | 0 | N | 1 |
| ARA-s | *Linyphiidae sp4* | 0 | 0 | 0 | 0 | 0 | 0 | 0 | 0 | 0 | 0 | 0 | 0 | 0 | 0 | 1 | N | 1 |
| ARA-s | *Linyphiidae sp5* | 0 | 0 | 1 | 0 | 0 | 0 | 0 | 0 | 0 | 0 | 0 | 0 | 0 | 0 | 0 | N | 1 |
| ARA-s | *Liocranidae sp1* | 0 | 0 | 0 | 0 | 0 | 0 | 0 | 0 | 0 | 0 | 0 | 4 | 1 | 1 | 0 | Y | 1 |
| ARA-s | *Liocranidae sp3* | 0 | 0 | 0 | 0 | 0 | 0 | 0 | 0 | 0 | 0 | 0 | 0 | 0 | 0 | 1 | N | 1 |
| ARA-s | *Liocranidae sp4* | 0 | 0 | 0 | 0 | 0 | 0 | 0 | 0 | 2 | 0 | 0 | 0 | 0 | 0 | 0 | N | 1 |
| ARA-s | *Micaria formicaria* | 0 | 0 | 0 | 0 | 0 | 0 | 0 | 2 | 1 | 0 | 0 | 0 | 0 | 0 | 0 | Y | 1 |
| ARA-s | *Neaetha membranosa* | 0 | 2 | 0 | 0 | 0 | 0 | 0 | 0 | 0 | 0 | 0 | 0 | 0 | 0 | 0 | N | 1 |
| ARA-s | *Nomisia sp* | 0 | 0 | 1 | 0 | 1 | 0 | 2 | 1 | 0 | 5 | 0 | 0 | 0 | 0 | 0 | Y | 1 |
| ARA-s | *Oonops sp1* | 0 | 0 | 0 | 0 | 0 | 0 | 0 | 1 | 0 | 0 | 0 | 0 | 0 | 0 | 0 | N | 1 |
| ARA-s | *Pardosa sp1* | 0 | 0 | 0 | 0 | 0 | 1 | 0 | 0 | 1 | 0 | 0 | 0 | 0 | 0 | 1 | Y | 1 |
| ARA-s | *Pardosa sp4* | 0 | 0 | 0 | 0 | 0 | 0 | 0 | 0 | 0 | 0 | 0 | 1 | 0 | 0 | 0 | N | 1 |
| ARA-s | *Phelgra fasciata* | 0 | 0 | 1 | 0 | 0 | 1 | 0 | 0 | 0 | 0 | 0 | 0 | 0 | 0 | 0 | N | 1 |
| ARA-s | *Pholcus opilionoides* | 0 | 0 | 0 | 0 | 0 | 0 | 0 | 0 | 0 | 0 | 0 | 0 | 0 | 1 | 0 | N | 1 |
| ARA-s | *Phrurolithus festivus* | 0 | 0 | 0 | 0 | 0 | 3 | 0 | 0 | 1 | 0 | 0 | 0 | 0 | 0 | 0 | Y | 1 |
| ARA-s | *Salticidae sp4* | 0 | 0 | 0 | 1 | 0 | 0 | 0 | 0 | 0 | 0 | 0 | 0 | 0 | 0 | 0 | N | 1 |
| ARA-s | *Selamia reticulata* | 0 | 0 | 0 | 0 | 0 | 0 | 0 | 0 | 0 | 0 | 0 | 0 | 1 | 0 | 0 | N | 1 |
| ARA-s | *Tegenaria fuesslinni* | 1 | 0 | 0 | 0 | 0 | 1 | 1 | 1 | 0 | 1 | 3 | 1 | 2 | 1 | 4 | Y | 1 |
| ARA-s | *Therididae sp1* | 0 | 0 | 1 | 1 | 0 | 0 | 0 | 1 | 0 | 0 | 0 | 0 | 0 | 0 | 0 | Y | 1 |
| ARA-s | *Therididae sp2* | 0 | 0 | 1 | 0 | 0 | 0 | 0 | 1 | 0 | 0 | 0 | 0 | 0 | 0 | 0 | N | 1 |
| ARA-s | *Zodarion pseudoelegans* | 1 | 0 | 0 | 0 | 1 | 1 | 3 | 1 | 0 | 0 | 1 | 1 | 1 | 0 | 0 | Y | 1 |
| ARA-s | *Zoropsis sp1* | 0 | 0 | 0 | 0 | 0 | 0 | 0 | 0 | 1 | 0 | 0 | 0 | 0 | 0 | 0 | N | 1 |
| BIR | *Aegithalos caudatus* | 0 | 0 | 0 | 0 | 0 | 0 | 0 | 0 | 0 | 0 | 0 | 0 | 2 | 0 | 0 | N | 1 |
| BIR | *Alectoris rufa* | 1 | 0 | 1 | 0 | 0 | 1 | 0 | 0 | 0 | 2 | 0 | 0 | 0 | 0 | 0 | Y | 2 |
| BIR | *Anthus campestris* | 0 | 0 | 0 | 0 | 0 | 0 | 1 | 0 | 1 | 0 | 0 | 0 | 0 | 0 | 0 | N | 2 |
| BIR | *Carduelis carduelis* | 0 | 0 | 0 | 0 | 0 | 0 | 0 | 0 | 0 | 0 | 2 | 0 | 0 | 0 | 0 | N | 2 |
| BIR | *Certhia brachydactyla* | 0 | 0 | 0 | 0 | 0 | 0 | 0 | 0 | 0 | 0 | 2 | 2 | 0 | 0 | 0 | Y | 1 |
| BIR | *Cettia cetti* | 0 | 0 | 0 | 0 | 0 | 0 | 0 | 0 | 0 | 0 | 0 | 0 | 2 | 0 | 1 | Y | 1 |
| BIR | *Columba palumbus* | 0 | 0 | 0 | 0 | 0 | 0 | 0 | 0 | 0 | 0 | 1 | 2 | 1 | 1 | 1 | Y | 2 |
| BIR | *Emberiza cirlus* | 0 | 0 | 0 | 0 | 0 | 0 | 0 | 0 | 0 | 0 | 1 | 0 | 0 | 0 | 0 | N | 2 |
| BIR | *Emberiza hortulana* | 0 | 0 | 0 | 0 | 0 | 2 | 1 | 0 | 2 | 2 | 0 | 0 | 0 | 0 | 0 | Y | 2 |
| BIR | *Erithacus rubecula* | 0 | 0 | 0 | 0 | 0 | 0 | 0 | 0 | 0 | 0 | 0 | 1 | 1 | 1 | 1 | Y | 1 |
| BIR | *Falco tinnunculus* | 0 | 0 | 0 | 0 | 0 | 0 | 0 | 0 | 1 | 0 | 0 | 0 | 0 | 0 | 0 | N | 1 |
| BIR | *Fringilla coelebs* | 0 | 0 | 0 | 0 | 0 | 0 | 0 | 0 | 0 | 0 | 0 | 1 | 0 | 0 | 1 | N | 2 |
| BIR | *Garrulus glandarius* | 0 | 0 | 0 | 0 | 0 | 0 | 0 | 0 | 1 | 0 | 1 | 0 | 0 | 0 | 1 | Y | 4 |
| BIR | *Hippolais polyglotta* | 0 | 0 | 0 | 1 | 0 | 1 | 1 | 1 | 0 | 0 | 1 | 0 | 0 | 0 | 0 | Y | 1 |
| BIR | *Lullula arborea* | 1 | 2 | 0 | 0 | 0 | 0 | 4 | 5 | 1 | 5 | 0 | 0 | 0 | 1 | 0 | Y | 2 |
| BIR | *Luscinia megarhynchos* | 0 | 0 | 1 | 0 | 0 | 0 | 0 | 0 | 0 | 0 | 1 | 0 | 2 | 1 | 0 | Y | 1 |
| BIR | *Parus caeruleus* | 0 | 0 | 0 | 0 | 0 | 0 | 0 | 0 | 0 | 0 | 0 | 1 | 0 | 0 | 0 | N | 1 |
| BIR | *Parus cristatus* | 0 | 0 | 0 | 2 | 0 | 0 | 0 | 0 | 0 | 0 | 1 | 0 | 3 | 0 | 3 | Y | 1 |
| BIR | *Parus major* | 0 | 0 | 0 | 0 | 0 | 0 | 0 | 0 | 0 | 0 | 4 | 0 | 0 | 0 | 2 | Y | 1 |
| BIR | *Phylloscopus bonelli* | 0 | 0 | 0 | 1 | 0 | 0 | 0 | 0 | 0 | 0 | 3 | 3 | 3 | 2 | 4 | Y | 1 |
| BIR | *Regulus ignicapillus* | 0 | 0 | 0 | 0 | 0 | 0 | 0 | 0 | 0 | 0 | 0 | 1 | 0 | 3 | 0 | Y | 1 |
| BIR | *Saxicola torquatus* | 1 | 0 | 3 | 0 | 2 | 0 | 4 | 1 | 3 | 1 | 0 | 0 | 0 | 0 | 0 | Y | 1 |
| BIR | *Serinus serinus* | 0 | 0 | 0 | 1 | 0 | 0 | 3 | 0 | 0 | 0 | 3 | 0 | 1 | 0 | 1 | Y | 2 |
| BIR | *Sylvia atricapilla* | 0 | 0 | 0 | 0 | 0 | 0 | 0 | 0 | 0 | 0 | 0 | 0 | 3 | 1 | 2 | Y | 1 |
| BIR | *Sylvia cantillans* | 1 | 4 | 2 | 2 | 2 | 1 | 3 | 2 | 3 | 0 | 2 | 0 | 0 | 2 | 0 | Y | 1 |
| BIR | *Sylvia undata* | 0 | 0 | 0 | 1 | 1 | 0 | 0 | 0 | 0 | 0 | 0 | 0 | 0 | 0 | 0 | N | 1 |
| BIR | *Troglodytes troglodytes* | 0 | 0 | 1 | 0 | 0 | 1 | 0 | 0 | 0 | 0 | 0 | 2 | 1 | 2 | 1 | Y | 1 |
| BIR | *Turdus merula* | 0 | 0 | 1 | 0 | 0 | 0 | 0 | 0 | 0 | 0 | 0 | 1 | 1 | 1 | 1 | Y | 1 |
| BIR | *Turdus viscivorus* | 0 | 0 | 0 | 1 | 0 | 0 | 0 | 0 | 0 | 0 | 0 | 0 | 0 | 0 | 0 | N | 4 |
| COL-s | *Blaps lusitanica* | 3 | 0 | 1 | 3 | 1 | 0 | 1 | 1 | 2 | 1 | 1 | 4 | 0 | 0 | 0 | Y | 3 |
| COL-s | *Cephennium thoracicum* | 0 | 0 | 0 | 0 | 0 | 0 | 0 | 0 | 0 | 0 | 0 | 0 | 1 | 0 | 0 | N | 3 |
| COL-s | *Dienerella elongata* | 0 | 0 | 0 | 0 | 0 | 0 | 1 | 0 | 0 | 0 | 0 | 1 | 0 | 0 | 3 | Y | 3 |
| COL-s | *Enicopus vittatus* | 0 | 4 | 0 | 1 | 3 | 1 | 0 | 1 | 3 | 0 | 0 | 0 | 0 | 0 | 0 | Y | 2 |
| COL-s | *Exosoma lusitanicum* | 0 | 0 | 0 | 0 | 1 | 0 | 0 | 0 | 0 | 0 | 0 | 0 | 0 | 0 | 0 | N | 2 |
| COL-s | *Harpalus attenuatus* | 0 | 1 | 0 | 0 | 0 | 0 | 0 | 0 | 0 | 1 | 0 | 0 | 0 | 0 | 0 | N | 2 |
| COL-s | *Hylastes attenuatus* | 0 | 0 | 0 | 0 | 0 | 0 | 0 | 0 | 0 | 0 | 2 | 0 | 0 | 0 | 0 | N | 2 |
| COL-s | *Hylurgus miklitzi* | 0 | 0 | 0 | 0 | 0 | 0 | 0 | 0 | 0 | 0 | 1 | 0 | 0 | 0 | 0 | N | 2 |
| COL-s | *Mantura rustica* | 1 | 0 | 0 | 0 | 0 | 0 | 0 | 1 | 3 | 0 | 0 | 0 | 0 | 0 | 0 | Y | 2 |
| COL-s | *Melanobaris morio* | 0 | 0 | 0 | 1 | 1 | 0 | 2 | 0 | 1 | 1 | 0 | 0 | 0 | 0 | 0 | Y | 2 |
| COL-s | *Microhoria fasciata* | 0 | 1 | 6 | 23 | 11 | 5 | 0 | 44 | 8 | 0 | 0 | 0 | 0 | 0 | 0 | Y | 3 |
| COL-s | *Nevraphes sp.* | 0 | 0 | 0 | 0 | 0 | 0 | 0 | 0 | 0 | 0 | 0 | 0 | 2 | 2 | 0 | Y | 3 |
| COL-s | *Ocypus olens* | 0 | 0 | 0 | 0 | 0 | 0 | 0 | 0 | 0 | 0 | 0 | 0 | 0 | 1 | 0 | N | 1 |
| COL-s | *Oryzaephilus surinamensis* | 0 | 0 | 0 | 1 | 0 | 0 | 0 | 0 | 0 | 0 | 0 | 0 | 0 | 0 | 0 | N | 3 |
| COL-s | *Oxythyrea funesta* | 0 | 0 | 0 | 0 | 0 | 0 | 2 | 0 | 0 | 0 | 0 | 0 | 0 | 0 | 0 | N | 3 |
| COL-s | *Phylan abbreviatus* | 0 | 1 | 0 | 0 | 1 | 0 | 0 | 1 | 5 | 0 | 0 | 0 | 0 | 0 | 0 | Y | 3 |
| COL-s | *Polydrusus confluens* | 0 | 0 | 0 | 0 | 0 | 1 | 1 | 0 | 0 | 0 | 0 | 0 | 0 | 0 | 0 | N | 2 |
| COL-s | *Ptinus timidus* | 0 | 0 | 0 | 0 | 0 | 0 | 0 | 0 | 0 | 0 | 1 | 0 | 0 | 0 | 0 | N | 3 |
| COL-s | *Scydmaenus ibericus* | 0 | 0 | 0 | 0 | 0 | 0 | 0 | 0 | 0 | 0 | 0 | 0 | 1 | 0 | 0 | N | 3 |
| COL-s | *Scymnus frontalis* | 0 | 2 | 0 | 0 | 0 | 0 | 0 | 1 | 0 | 0 | 0 | 0 | 0 | 0 | 0 | Y | 1 |
| COL-s | *Sepedophilus testaceus* | 0 | 0 | 0 | 0 | 0 | 0 | 0 | 0 | 0 | 0 | 0 | 0 | 1 | 1 | 0 | N | 3 |
| COL-s | *Sericoderus pecirkanus* | 0 | 0 | 0 | 0 | 0 | 1 | 0 | 0 | 2 | 0 | 0 | 0 | 1 | 0 | 0 | Y | 3 |
| COL-s | *Staphylinidae sp1* | 0 | 0 | 0 | 0 | 0 | 0 | 0 | 0 | 0 | 0 | 1 | 0 | 0 | 0 | 0 | N | 1 |
| COL-s | *Staphylinidae sp2* | 0 | 0 | 0 | 0 | 1 | 0 | 0 | 0 | 0 | 0 | 3 | 0 | 0 | 2 | 0 | Y | 1 |
| COL-s | *Staphylinidae sp3* | 0 | 0 | 0 | 0 | 0 | 0 | 0 | 0 | 1 | 0 | 0 | 0 | 0 | 0 | 0 | N | 1 |
| COL-s | *Staphylinidae sp4* | 1 | 0 | 0 | 0 | 0 | 0 | 1 | 0 | 0 | 0 | 0 | 0 | 0 | 0 | 0 | N | 1 |
| COL-s | *Tachyporus nitidulus* | 0 | 0 | 0 | 0 | 0 | 0 | 0 | 0 | 0 | 0 | 1 | 0 | 0 | 0 | 0 | N | 3 |
| COL-v | *Acmaeodera nigellata* | 0 | 0 | 1 | 0 | 0 | 0 | 0 | 0 | 0 | 0 | 0 | 0 | 0 | 0 | 0 | N | 2 |
| COL-v | *Agapanthia cardui* | 1 | 0 | 0 | 0 | 0 | 0 | 0 | 0 | 0 | 0 | 0 | 0 | 0 | 0 | 0 | N | 2 |
| COL-v | *Agrilus hyperici* | 0 | 1 | 0 | 0 | 1 | 0 | 0 | 0 | 0 | 1 | 0 | 0 | 0 | 0 | 0 | Y | 2 |
| COL-v | *Anthaxia godeti* | 0 | 0 | 0 | 0 | 2 | 0 | 0 | 0 | 1 | 0 | 3 | 0 | 0 | 0 | 0 | Y | 2 |
| COL-v | *Aphthona lutescens* | 7 | 6 | 0 | 1 | 17 | 0 | 0 | 1 | 39 | 0 | 0 | 0 | 0 | 0 | 1 | Y | 2 |
| COL-v | *Aplocnemus virens* | 0 | 0 | 0 | 0 | 0 | 0 | 0 | 0 | 2 | 0 | 0 | 0 | 0 | 0 | 0 | N | 1 |
| COL-v | *Aredolpona cordigera* | 0 | 0 | 0 | 0 | 0 | 0 | 0 | 0 | 0 | 0 | 0 | 1 | 0 | 0 | 0 | N | 2 |
| COL-v | *Arthrolips convexiuscula* | 1 | 0 | 0 | 0 | 0 | 0 | 0 | 1 | 0 | 0 | 0 | 0 | 0 | 0 | 0 | N | 3 |
| COL-v | *Athous godarti* | 0 | 0 | 0 | 0 | 0 | 0 | 0 | 0 | 1 | 0 | 0 | 2 | 0 | 1 | 0 | Y | 2 |
| COL-v | *Attalus amictus* | 0 | 0 | 0 | 0 | 0 | 0 | 0 | 0 | 0 | 0 | 0 | 3 | 0 | 0 | 0 | Y | 2 |
| COL-v | *Attalus pictus* | 0 | 0 | 0 | 0 | 0 | 0 | 0 | 0 | 0 | 0 | 0 | 1 | 1 | 1 | 0 | Y | 2 |
| COL-v | *Axinotarsus marginalis* | 0 | 0 | 0 | 0 | 0 | 0 | 0 | 0 | 0 | 0 | 1 | 3 | 3 | 1 | 0 | Y | 1 |
| COL-v | *Calomicrus circumfusus* | 0 | 0 | 0 | 0 | 0 | 0 | 0 | 0 | 0 | 0 | 0 | 0 | 0 | 0 | 1 | N | 2 |
| COL-v | *Charopus pallipes* | 0 | 0 | 3 | 0 | 0 | 0 | 0 | 1 | 0 | 0 | 0 | 0 | 0 | 0 | 0 | Y | 2 |
| COL-v | *Chrysolina americana* | 0 | 0 | 0 | 0 | 1 | 0 | 0 | 1 | 0 | 0 | 1 | 0 | 3 | 2 | 5 | Y | 2 |
| COL-v | *Coccinella septempunctata* | 1 | 0 | 1 | 0 | 0 | 0 | 0 | 0 | 0 | 0 | 0 | 0 | 0 | 0 | 0 | N | 1 |
| COL-v | *Colotes javeti* | 0 | 0 | 0 | 0 | 0 | 0 | 0 | 0 | 0 | 0 | 2 | 0 | 0 | 0 | 0 | N | 2 |
| COL-v | *Colotes maculatus* | 0 | 0 | 0 | 0 | 0 | 0 | 2 | 0 | 0 | 0 | 0 | 0 | 0 | 0 | 1 | Y | 2 |
| COL-v | *Coptocephala scopolina* | 1 | 0 | 0 | 1 | 0 | 0 | 0 | 0 | 0 | 1 | 0 | 0 | 0 | 0 | 0 | Y | 2 |
| COL-v | *Cryptocephalus nitidulus* | 0 | 0 | 0 | 0 | 0 | 2 | 1 | 2 | 2 | 0 | 0 | 0 | 0 | 0 | 0 | Y | 2 |
| COL-v | *Cryptocephalus ramburii* | 1 | 0 | 0 | 0 | 0 | 0 | 0 | 0 | 0 | 1 | 0 | 0 | 0 | 0 | 0 | N | 2 |
| COL-v | *Cryptocephalus sulphureus* | 0 | 0 | 0 | 0 | 0 | 0 | 0 | 0 | 0 | 1 | 0 | 0 | 0 | 0 | 0 | N | 2 |
| COL-v | *Danacaea longiceps* | 0 | 0 | 5 | 1 | 4 | 0 | 21 | 1 | 4 | 0 | 0 | 0 | 0 | 0 | 0 | Y | 4 |
| COL-v | *Dasytes subaeneus* | 0 | 0 | 1 | 0 | 0 | 0 | 0 | 0 | 0 | 0 | 0 | 0 | 0 | 0 | 0 | N | 2 |
| COL-v | *Dicladispa testacea* | 0 | 0 | 0 | 0 | 0 | 0 | 0 | 0 | 1 | 2 | 0 | 0 | 0 | 0 | 0 | Y | 2 |
| COL-v | *Enicopus vittatus* | 0 | 0 | 0 | 1 | 0 | 1 | 1 | 0 | 3 | 1 | 0 | 0 | 0 | 0 | 0 | Y | 3 |
| COL-v | *Evaniocera duforti* | 0 | 0 | 0 | 0 | 0 | 0 | 1 | 0 | 0 | 0 | 0 | 0 | 0 | 0 | 0 | N | 1 |
| COL-v | *Exosoma lusitanicum* | 0 | 0 | 0 | 0 | 0 | 0 | 0 | 0 | 0 | 0 | 0 | 0 | 0 | 1 | 0 | N | 2 |
| COL-v | *Harpalus attenuatus* | 0 | 1 | 0 | 0 | 0 | 0 | 0 | 0 | 0 | 0 | 0 | 0 | 0 | 0 | 0 | N | 2 |
| COL-v | *Labidostomis lusitanica* | 0 | 0 | 0 | 0 | 1 | 0 | 0 | 0 | 0 | 0 | 0 | 0 | 0 | 0 | 0 | N | 2 |
| COL-v | *Lachnaia pubescens* | 0 | 1 | 1 | 0 | 0 | 1 | 1 | 0 | 0 | 0 | 0 | 0 | 0 | 0 | 0 | Y | 2 |
| COL-v | *Lagria hirta* | 0 | 0 | 0 | 0 | 0 | 0 | 0 | 0 | 0 | 1 | 0 | 0 | 0 | 0 | 1 | N | 2 |
| COL-v | *Lamprias rufipes* | 0 | 0 | 0 | 0 | 0 | 0 | 0 | 0 | 0 | 1 | 0 | 0 | 0 | 0 | 0 | N | 1 |
| COL-v | *Lasioderma serricornis* | 0 | 0 | 0 | 0 | 0 | 0 | 0 | 0 | 0 | 0 | 0 | 0 | 0 | 0 | 1 | N | 3 |
| COL-v | *Lasiorhynchites coeruleocephalus* | 0 | 0 | 0 | 0 | 0 | 0 | 0 | 0 | 0 | 0 | 2 | 0 | 0 | 0 | 0 | N | 2 |
| COL-v | *Lobonix aeneus* | 0 | 0 | 0 | 0 | 1 | 0 | 0 | 0 | 0 | 0 | 0 | 0 | 0 | 0 | 0 | N | 2 |
| COL-v | *Longitarsus pellucidus* | 0 | 10 | 0 | 0 | 1 | 0 | 0 | 0 | 7 | 1 | 0 | 1 | 0 | 0 | 0 | Y | 2 |
| COL-v | *Malthinus seriepunctatus* | 0 | 0 | 0 | 0 | 0 | 0 | 0 | 0 | 0 | 0 | 0 | 1 | 0 | 1 | 0 | N | 3 |
| COL-v | *Mantura rustica* | 3 | 0 | 0 | 1 | 2 | 2 | 0 | 9 | 8 | 1 | 4 | 0 | 0 | 0 | 0 | Y | 2 |
| COL-v | *Melanobaris morio* | 0 | 0 | 0 | 0 | 0 | 0 | 0 | 0 | 0 | 1 | 0 | 0 | 0 | 0 | 0 | N | 2 |
| COL-v | *Melanophthalma taurica* | 0 | 0 | 0 | 0 | 0 | 0 | 0 | 0 | 0 | 0 | 0 | 0 | 0 | 0 | 2 | N | 3 |
| COL-v | *Meliboeus aeratus* | 0 | 0 | 0 | 0 | 0 | 0 | 0 | 0 | 0 | 0 | 0 | 0 | 0 | 1 | 0 | N | 2 |
| COL-v | *Microhoria fasciata* | 7 | 0 | 3 | 0 | 2 | 0 | 0 | 0 | 3 | 1 | 1 | 0 | 0 | 0 | 2 | Y | 3 |
| COL-v | *Mordellistena sp.* | 3 | 2 | 10 | 0 | 2 | 3 | 0 | 1 | 23 | 2 | 0 | 0 | 0 | 0 | 0 | Y | 2 |
| COL-v | *Mycterus curculioides* | 0 | 0 | 3 | 0 | 0 | 0 | 0 | 2 | 0 | 0 | 0 | 0 | 0 | 0 | 0 | Y | 2 |
| COL-v | *Mylabris quadripunctata* | 1 | 0 | 0 | 1 | 0 | 0 | 0 | 0 | 0 | 0 | 0 | 0 | 0 | 0 | 0 | N | 2 |
| COL-v | *Oedemera barbara* | 0 | 0 | 0 | 0 | 0 | 1 | 0 | 0 | 0 | 0 | 0 | 0 | 0 | 0 | 1 | N | 2 |
| COL-v | *Oedemera lateralis* | 0 | 1 | 0 | 1 | 0 | 0 | 0 | 6 | 1 | 0 | 0 | 0 | 0 | 0 | 0 | Y | 2 |
| COL-v | *Omiamima concinna* | 0 | 3 | 1 | 0 | 1 | 5 | 5 | 3 | 0 | 7 | 0 | 1 | 0 | 0 | 1 | Y | 2 |
| COL-v | *Pachrybrachis antigae* | 0 | 0 | 0 | 1 | 2 | 0 | 0 | 0 | 0 | 0 | 0 | 0 | 0 | 0 | 0 | Y | 2 |
| COL-v | *Phyllotreta vittula* | 0 | 0 | 0 | 0 | 0 | 0 | 0 | 6 | 1 | 0 | 0 | 0 | 0 | 0 | 0 | Y | 2 |
| COL-v | *Podagrica fuscicornis* | 0 | 0 | 0 | 0 | 0 | 0 | 0 | 4 | 0 | 0 | 0 | 0 | 0 | 0 | 0 | Y | 2 |
| COL-v | *Polydrusus confluens* | 8 | 5 | 2 | 28 | 45 | 10 | 7 | 0 | 0 | 2 | 0 | 0 | 0 | 0 | 1 | Y | 2 |
| COL-v | *Psilothrix viridicoerulea* | 0 | 0 | 0 | 0 | 0 | 0 | 0 | 2 | 0 | 0 | 0 | 0 | 0 | 0 | 0 | N | 4 |
| COL-v | *Rhyzobius chrysomeloides* | 0 | 0 | 0 | 1 | 0 | 0 | 0 | 1 | 0 | 0 | 0 | 0 | 0 | 0 | 1 | Y | 1 |
| COL-v | *Scraptia dubia* | 3 | 1 | 5 | 0 | 2 | 1 | 0 | 3 | 3 | 0 | 0 | 5 | 0 | 1 | 0 | Y | 2 |
| COL-v | *Scymnus frontalis* | 7 | 1 | 4 | 1 | 2 | 0 | 1 | 1 | 2 | 0 | 0 | 0 | 0 | 0 | 0 | Y | 1 |
| COL-v | *Scymnus interruptus* | 0 | 0 | 0 | 0 | 0 | 0 | 0 | 0 | 0 | 0 | 0 | 0 | 1 | 0 | 0 | N | 1 |
| COL-v | *Scymnus suturalis* | 1 | 0 | 0 | 0 | 0 | 0 | 0 | 1 | 0 | 0 | 0 | 0 | 0 | 0 | 0 | N | 1 |
| COL-v | *Sitona macularis* | 0 | 0 | 0 | 0 | 0 | 0 | 0 | 0 | 7 | 0 | 0 | 0 | 1 | 0 | 0 | Y | 2 |
| COL-v | *Spermophagus sericeus* | 0 | 1 | 0 | 0 | 11 | 0 | 1 | 0 | 6 | 0 | 0 | 0 | 0 | 0 | 0 | Y | 2 |
| COL-v | *Staphylinidae sp.* | 0 | 0 | 0 | 0 | 0 | 0 | 0 | 1 | 0 | 0 | 0 | 1 | 0 | 0 | 0 | N | 1 |
| COL-v | *Trichodes leucopsideus* | 0 | 0 | 0 | 0 | 0 | 0 | 0 | 0 | 1 | 1 | 0 | 0 | 0 | 0 | 0 | N | 1 |
| COL-v | *Tychius argentatus* | 0 | 0 | 7 | 7 | 1 | 0 | 0 | 1 | 7 | 6 | 0 | 1 | 1 | 0 | 0 | Y | 2 |
| FOR-s | *Aphaenogaster gibbosa* | 20 | 4 | 0 | 0 | 0 | 0 | 1 | 0 | 0 | 0 | 0 | 1 | 11 | 0 | 0 | Y | 4 |
| FOR-s | *Aphaenogaster subterranea* | 0 | 0 | 0 | 0 | 0 | 0 | 0 | 0 | 0 | 0 | 0 | 0 | 0 | 0 | 3 | Y | 4 |
| FOR-s | *Camponotus cruentatus* | 0 | 0 | 0 | 0 | 7 | 0 | 0 | 0 | 0 | 1 | 0 | 0 | 0 | 0 | 0 | Y | 4 |
| FOR-s | *Camponotus lateralis* | 0 | 0 | 0 | 0 | 0 | 0 | 0 | 0 | 0 | 0 | 0 | 1 | 0 | 0 | 0 | N | 4 |
| FOR-s | *Camponotus piceus* | 3 | 0 | 0 | 1 | 0 | 0 | 0 | 0 | 0 | 0 | 0 | 0 | 0 | 0 | 0 | Y | 4 |
| FOR-s | *Camponotus pilicornis* | 1 | 1 | 0 | 1 | 1 | 1 | 1 | 1 | 0 | 0 | 4 | 1 | 1 | 3 | 0 | Y | 4 |
| FOR-s | *Camponotus sylvaticus* | 2 | 0 | 0 | 0 | 0 | 0 | 0 | 2 | 1 | 0 | 0 | 0 | 1 | 4 | 0 | Y | 4 |
| FOR-s | *Chalepoxenus kutteri* | 0 | 0 | 0 | 0 | 0 | 0 | 0 | 0 | 0 | 0 | 0 | 0 | 1 | 0 | 0 | N | 4 |
| FOR-s | *Crematogaster scutellaris* | 0 | 0 | 0 | 0 | 0 | 0 | 0 | 0 | 0 | 0 | 0 | 4 | 2 | 1 | 8 | Y | 4 |
| FOR-s | *Formica gagates* | 0 | 0 | 0 | 0 | 0 | 0 | 0 | 0 | 1 | 0 | 0 | 5 | 0 | 0 | 1 | Y | 4 |
| FOR-s | *Formica gerardi* | 0 | 0 | 1 | 1 | 0 | 2 | 1 | 0 | 14 | 0 | 0 | 0 | 0 | 2 | 5 | Y | 4 |
| FOR-s | *Formica subrufa* | 0 | 0 | 0 | 0 | 0 | 0 | 29 | 0 | 4 | 0 | 0 | 0 | 2 | 2 | 0 | Y | 4 |
| FOR-s | *Lasius cinereus* | 0 | 0 | 1 | 1 | 0 | 15 | 1 | 0 | 73 | 0 | 22 | 0 | 0 | 0 | 0 | Y | 4 |
| FOR-s | *Lasius grandis* | 0 | 0 | 0 | 2 | 0 | 163 | 2 | 1 | 2 | 25 | 38 | 0 | 40 | 1 | 0 | Y | 4 |
| FOR-s | *Leptanilla revelierii* | 0 | 0 | 0 | 0 | 0 | 0 | 0 | 0 | 0 | 1 | 0 | 0 | 0 | 0 | 0 | N | 1 |
| FOR-s | *Myrmica specioides* | 0 | 1 | 0 | 0 | 0 | 0 | 0 | 0 | 0 | 0 | 1 | 29 | 0 | 0 | 0 | Y | 4 |
| FOR-s | *Myrmica spinosior* | 0 | 1 | 0 | 0 | 0 | 0 | 0 | 0 | 0 | 0 | 0 | 6 | 32 | 0 | 4 | Y | 4 |
| FOR-s | *Pheidole pallidula* | 36 | 2 | 165 | 214 | 56 | 0 | 3 | 47 | 30 | 24 | 83 | 18 | 21 | 55 | 0 | Y | 4 |
| FOR-s | *Plagiolepis pygmaea* | 4 | 3 | 8 | 23 | 8 | 2 | 4 | 12 | 2 | 3 | 9 | 11 | 2 | 8 | 1 | Y | 2 |
| FOR-s | *Plagiolepis xene* | 0 | 0 | 0 | 0 | 0 | 1 | 0 | 0 | 0 | 0 | 0 | 0 | 0 | 1 | 0 | N | 2 |
| FOR-s | *Polyergus rufescens* | 0 | 0 | 0 | 0 | 0 | 0 | 0 | 0 | 0 | 0 | 0 | 0 | 1 | 0 | 0 | N | 4 |
| FOR-s | *Solenopsis sp* | 2 | 0 | 0 | 3 | 0 | 0 | 1 | 0 | 0 | 0 | 8 | 0 | 2 | 3 | 0 | Y | 1 |
| FOR-s | *Strongylognatus testaceus* | 0 | 1 | 0 | 0 | 0 | 0 | 0 | 0 | 0 | 0 | 0 | 0 | 0 | 0 | 0 | N | 4 |
| FOR-s | *Tapinoma ambiguum* | 0 | 15 | 12 | 14 | 0 | 7 | 0 | 5 | 1 | 0 | 0 | 0 | 0 | 0 | 0 | Y | 2 |
| FOR-s | *Tapinoma nigerrimum* | 361 | 0 | 0 | 0 | 2 | 10 | 0 | 352 | 2 | 0 | 0 | 4 | 0 | 0 | 3 | Y | 4 |
| FOR-s | *Temnothorax gredosi* | 0 | 0 | 0 | 0 | 0 | 0 | 0 | 0 | 0 | 0 | 0 | 1 | 0 | 0 | 0 | N | 4 |
| FOR-s | *Temnothorax lichtensteini* | 0 | 0 | 0 | 0 | 0 | 0 | 0 | 0 | 0 | 0 | 0 | 3 | 4 | 0 | 1 | Y | 4 |
| FOR-s | *Tetramorium caespitum* | 0 | 2 | 0 | 1 | 0 | 11 | 36 | 4 | 6 | 0 | 0 | 0 | 2 | 0 | 0 | Y | 4 |
| FOR-s | *Tetramorium forte* | 0 | 98 | 0 | 0 | 0 | 0 | 0 | 0 | 0 | 0 | 0 | 0 | 0 | 0 | 0 | Y | 4 |
| FOR-s | *Tetramorium impurum* | 0 | 0 | 7 | 1 | 0 | 0 | 0 | 0 | 0 | 0 | 0 | 0 | 0 | 0 | 0 | Y | 4 |
| FOR-v | *Camponotus lateralis* | 0 | 1 | 0 | 0 | 0 | 0 | 0 | 0 | 0 | 0 | 0 | 5 | 1 | 0 | 3 | Y | 4 |
| FOR-v | *Camponotus piceus* | 3 | 2 | 4 | 1 | 2 | 2 | 2 | 0 | 0 | 5 | 1 | 0 | 6 | 2 | 2 | Y | 4 |
| FOR-v | *Camponotus pilicornis* | 0 | 0 | 0 | 0 | 1 | 0 | 0 | 0 | 0 | 0 | 0 | 0 | 0 | 0 | 0 | N | 4 |
| FOR-v | *Crematogaster scutellaris* | 0 | 0 | 0 | 0 | 0 | 0 | 0 | 0 | 0 | 0 | 1 | 1 | 0 | 0 | 0 | N | 4 |
| FOR-v | *Formica gagates* | 0 | 0 | 0 | 0 | 0 | 0 | 0 | 0 | 0 | 0 | 0 | 2 | 0 | 0 | 3 | Y | 4 |
| FOR-v | *Formica gerardi* | 0 | 0 | 2 | 0 | 0 | 1 | 0 | 0 | 5 | 1 | 0 | 4 | 0 | 1 | 0 | Y | 4 |
| FOR-v | *Formica subrufa* | 1 | 0 | 0 | 0 | 0 | 0 | 2 | 0 | 0 | 0 | 0 | 0 | 0 | 0 | 0 | Y | 4 |
| FOR-v | *Lasius cinereus* | 0 | 0 | 0 | 0 | 2 | 0 | 0 | 2 | 2 | 2 | 5 | 0 | 0 | 0 | 0 | Y | 4 |
| FOR-v | *Lasius grandis* | 0 | 0 | 4 | 0 | 0 | 1 | 0 | 0 | 0 | 0 | 0 | 0 | 1 | 0 | 0 | Y | 4 |
| FOR-v | *Myrmica spinosior* | 0 | 0 | 0 | 0 | 0 | 0 | 0 | 0 | 0 | 0 | 0 | 0 | 1 | 0 | 2 | Y | 4 |
| FOR-v | *Plagiolepis pygmaea* | 20 | 13 | 13 | 1 | 3 | 2 | 8 | 8 | 10 | 31 | 35 | 24 | 51 | 26 | 6 | Y | 2 |
| FOR-v | *Tapinoma ambiguum* | 0 | 6 | 1 | 0 | 0 | 0 | 0 | 0 | 0 | 0 | 0 | 0 | 0 | 0 | 0 | Y | 4 |
| FOR-v | *Tapinoma nigerrimum* | 52 | 7 | 1 | 0 | 1 | 0 | 0 | 5 | 0 | 1 | 0 | 0 | 0 | 0 | 0 | Y | 4 |
| FOR-v | *Temnothorax niger* | 0 | 1 | 0 | 0 | 0 | 0 | 0 | 0 | 0 | 0 | 0 | 0 | 6 | 0 | 0 | Y | 4 |
| FOR-v | *Temnothorax parvulus* | 0 | 0 | 0 | 0 | 0 | 0 | 0 | 0 | 0 | 0 | 0 | 4 | 0 | 0 | 0 | Y | 4 |
| FOR-v | *Temnothorax rabaudi* | 0 | 0 | 0 | 0 | 0 | 0 | 0 | 0 | 0 | 0 | 0 | 2 | 0 | 0 | 6 | Y | 4 |
| FOR-v | *Temnothorax racovitzai* | 0 | 0 | 1 | 0 | 0 | 0 | 0 | 0 | 0 | 0 | 0 | 0 | 0 | 0 | 0 | N | 4 |
| FOR-v | *Tetramorium forte* | 0 | 2 | 0 | 0 | 0 | 0 | 0 | 0 | 0 | 0 | 0 | 0 | 0 | 0 | 0 | N | 4 |
| GAS | *Abida polyodon* | 0 | 2 | 0 | 0 | 0 | 0 | 0 | 0 | 0 | 0 | 2 | 1 | 4 | 0 | 12 | Y | 2 |
| GAS | *Cecilioides acicula* | 0 | 0 | 0 | 0 | 0 | 0 | 0 | 0 | 0 | 0 | 1 | 0 | 0 | 0 | 0 | N | 3 |
| GAS | *Cepaea nemoralis* | 0 | 0 | 0 | 2 | 0 | 0 | 0 | 0 | 0 | 0 | 0 | 3 | 2 | 0 | 1 | Y | 2 |
| GAS | *Cernuella virgata* | 0 | 0 | 0 | 2 | 0 | 1 | 0 | 0 | 1 | 0 | 0 | 0 | 0 | 0 | 0 | Y | 2 |
| GAS | *Cornu aspersum* | 0 | 6 | 2 | 3 | 0 | 0 | 0 | 0 | 0 | 0 | 0 | 0 | 0 | 1 | 6 | Y | 2 |
| GAS | *Deroceras altimirai* | 0 | 2 | 2 | 0 | 0 | 0 | 0 | 0 | 0 | 0 | 0 | 1 | 0 | 0 | 0 | Y | 3 |
| GAS | *Euconulus fulvus* | 0 | 0 | 0 | 0 | 0 | 0 | 0 | 0 | 0 | 0 | 0 | 4 | 0 | 0 | 4 | Y | 2 |
| GAS | *Ferussacia folliculus* | 0 | 0 | 0 | 0 | 0 | 0 | 0 | 0 | 0 | 0 | 0 | 0 | 0 | 2 | 0 | N | 2 |
| GAS | *Helicigona lapicida* | 0 | 0 | 0 | 0 | 0 | 0 | 1 | 0 | 0 | 0 | 0 | 3 | 1 | 0 | 1 | Y | 2 |
| GAS | *Jaminia quadridens* | 0 | 0 | 0 | 0 | 0 | 0 | 0 | 0 | 0 | 0 | 0 | 0 | 3 | 0 | 0 | Y | 2 |
| GAS | *Monacha cartusiana* | 0 | 1 | 0 | 1 | 0 | 0 | 0 | 0 | 0 | 0 | 0 | 0 | 0 | 5 | 0 | Y | 2 |
| GAS | *Montserratina bofilliana* | 0 | 0 | 0 | 0 | 0 | 0 | 0 | 1 | 0 | 0 | 0 | 0 | 0 | 0 | 0 | N | 2 |
| GAS | *Otala punctata* | 0 | 0 | 0 | 1 | 0 | 0 | 0 | 0 | 0 | 0 | 3 | 0 | 0 | 1 | 0 | Y | 2 |
| GAS | *Oxychilus courquini* | 0 | 0 | 0 | 0 | 0 | 0 | 0 | 1 | 0 | 0 | 0 | 0 | 1 | 1 | 0 | Y | 1 |
| GAS | *Oxychilus draparnaudi* | 0 | 0 | 0 | 1 | 0 | 0 | 0 | 0 | 0 | 0 | 0 | 0 | 0 | 1 | 1 | Y | 1 |
| GAS | *Paralaoma servilis* | 0 | 0 | 0 | 0 | 0 | 0 | 0 | 0 | 0 | 0 | 0 | 1 | 0 | 2 | 0 | Y | 2 |
| GAS | *Pomatias elegans* | 0 | 1 | 0 | 0 | 0 | 0 | 0 | 0 | 0 | 0 | 7 | 10 | 16 | 5 | 10 | Y | 2 |
| GAS | *Pseudotachea splendida* | 0 | 0 | 2 | 0 | 0 | 0 | 0 | 0 | 0 | 0 | 1 | 0 | 0 | 7 | 3 | Y | 2 |
| GAS | *Punctum pygmaeum* | 0 | 0 | 0 | 0 | 0 | 0 | 0 | 0 | 0 | 0 | 0 | 0 | 0 | 0 | 1 | N | 2 |
| GAS | *Rumina decollate* | 0 | 0 | 0 | 0 | 0 | 0 | 0 | 0 | 0 | 0 | 0 | 0 | 1 | 0 | 0 | N | 3 |
| GAS | *Truncatellina callicratis* | 0 | 0 | 0 | 0 | 0 | 0 | 0 | 0 | 0 | 0 | 1 | 1 | 0 | 0 | 0 | N | 2 |
| GAS | *Vallonia costata* | 0 | 0 | 0 | 0 | 0 | 0 | 0 | 0 | 0 | 0 | 0 | 0 | 1 | 0 | 0 | N | 2 |
| GAS | *Vitrea sp* | 0 | 0 | 0 | 0 | 0 | 0 | 0 | 0 | 0 | 0 | 0 | 0 | 1 | 0 | 0 | N | 3 |
| GAS | *Xerocrassa montserratensis* | 0 | 0 | 0 | 0 | 0 | 0 | 1 | 0 | 0 | 3 | 1 | 0 | 1 | 0 | 0 | Y | 2 |
| GAS | *Xerocrassa penchinati* | 30 | 6 | 27 | 19 | 1 | 11 | 1 | 24 | 1 | 5 | 4 | 1 | 0 | 5 | 0 | Y | 2 |
| GAS | *Xerosecta arigonis* | 0 | 0 | 0 | 4 | 0 | 0 | 0 | 0 | 0 | 0 | 0 | 0 | 0 | 12 | 0 | Y | 2 |
| HET-s | *Beosus maritimus* | 0 | 0 | 0 | 0 | 0 | 0 | 0 | 6 | 0 | 1 | 0 | 0 | 0 | 0 | 0 | Y | 2 |
| HET-s | *Bothrostethus annulipes* | 1 | 0 | 0 | 0 | 0 | 0 | 1 | 0 | 0 | 0 | 0 | 0 | 0 | 0 | 0 | N | 2 |
| HET-s | *Coranus griseus* | 1 | 0 | 0 | 0 | 0 | 0 | 0 | 0 | 0 | 0 | 0 | 0 | 0 | 0 | 0 | N | 1 |
| HET-s | *Cydnus aterrimus* | 0 | 0 | 0 | 0 | 0 | 0 | 0 | 0 | 5 | 0 | 0 | 0 | 0 | 0 | 0 | Y | 2 |
| HET-s | *Emblethis duplicatus* | 0 | 0 | 0 | 0 | 0 | 0 | 0 | 2 | 0 | 0 | 0 | 0 | 0 | 0 | 0 | N | 2 |
| HET-s | *Geotomus punctulatus* | 0 | 1 | 0 | 0 | 0 | 0 | 0 | 0 | 0 | 0 | 0 | 0 | 0 | 0 | 0 | N | 2 |
| HET-s | *Ischnocoris angustulus* | 0 | 0 | 0 | 0 | 0 | 0 | 0 | 1 | 0 | 0 | 0 | 0 | 0 | 0 | 0 | N | 2 |
| HET-s | *Lasiocoris anomalus* | 0 | 1 | 0 | 0 | 0 | 0 | 0 | 0 | 0 | 0 | 0 | 0 | 0 | 0 | 0 | N | 2 |
| HET-s | *Leptopus marmoratus* | 0 | 0 | 0 | 0 | 0 | 0 | 1 | 0 | 0 | 0 | 0 | 0 | 0 | 0 | 0 | N | 1 |
| HET-s | *Lygaeosoma sardeum* | 0 | 0 | 14 | 0 | 0 | 0 | 0 | 0 | 3 | 0 | 0 | 0 | 0 | 0 | 0 | Y | 2 |
| HET-s | *Megalonotus sabulicola* | 0 | 1 | 0 | 0 | 0 | 0 | 0 | 0 | 0 | 0 | 0 | 0 | 0 | 0 | 0 | N | 2 |
| HET-s | *Melanocoryphus albomaculatus* | 0 | 0 | 0 | 0 | 1 | 0 | 0 | 2 | 0 | 0 | 0 | 0 | 0 | 0 | 0 | Y | 2 |
| HET-s | *Odontoscelis fuliginosa* | 0 | 1 | 0 | 0 | 0 | 0 | 0 | 0 | 7 | 0 | 0 | 0 | 0 | 0 | 0 | Y | 2 |
| HET-s | *Odontoscelis lineola* | 1 | 0 | 0 | 0 | 0 | 0 | 0 | 0 | 1 | 0 | 0 | 0 | 0 | 0 | 0 | N | 2 |
| HET-s | *Peirates stridulus* | 0 | 0 | 0 | 0 | 0 | 1 | 0 | 0 | 0 | 0 | 0 | 0 | 0 | 0 | 0 | N | 1 |
| HET-s | *Phytocoris vittiger* | 0 | 0 | 0 | 0 | 1 | 0 | 0 | 0 | 0 | 0 | 0 | 0 | 0 | 0 | 0 | N | 2 |
| HET-s | *Plinthisus magnieni* | 0 | 0 | 0 | 0 | 0 | 0 | 0 | 0 | 0 | 0 | 0 | 0 | 4 | 0 | 0 | Y | 2 |
| HET-s | *Ploiaria putoni* | 0 | 0 | 0 | 0 | 0 | 0 | 0 | 0 | 0 | 0 | 0 | 1 | 2 | 0 | 18 | Y | 1 |
| HET-s | *Rhynocoris cuspidatus* | 0 | 1 | 0 | 0 | 0 | 1 | 2 | 0 | 1 | 0 | 0 | 0 | 0 | 0 | 0 | Y | 1 |
| HET-s | *Tropistethus holosericeus* | 0 | 0 | 0 | 0 | 1 | 0 | 0 | 0 | 0 | 0 | 0 | 0 | 0 | 0 | 0 | N | 2 |
| ORT-s | *Barbitistes fischeri* | 0 | 0 | 0 | 0 | 1 | 1 | 0 | 0 | 0 | 0 | 1 | 0 | 0 | 2 | 0 | Y | 4 |
| ORT-s | *Calliptamus barbarus* | 1 | 1 | 1 | 2 | 5 | 0 | 0 | 1 | 7 | 5 | 4 | 1 | 2 | 4 | 0 | Y | 2 |
| ORT-s | *Decticus albifrons* | 0 | 0 | 0 | 0 | 0 | 0 | 0 | 0 | 0 | 1 | 0 | 0 | 0 | 0 | 0 | N | 4 |
| ORT-s | *Euchorthippus chopardi* | 0 | 1 | 0 | 1 | 0 | 0 | 0 | 0 | 0 | 0 | 4 | 0 | 4 | 0 | 0 | Y | 2 |
| ORT-s | *Eugryllodes pipiens* | 1 | 0 | 0 | 0 | 0 | 0 | 0 | 0 | 0 | 0 | 0 | 0 | 0 | 0 | 0 | N | 4 |
| ORT-s | *Oedipoda caerulescens* | 1 | 0 | 0 | 0 | 0 | 0 | 0 | 0 | 0 | 0 | 0 | 0 | 0 | 0 | 0 | N | 2 |
| ORT-s | *Phaneroptera nana* | 1 | 1 | 2 | 0 | 3 | 2 | 0 | 0 | 0 | 1 | 2 | 8 | 1 | 4 | 1 | Y | 4 |
| ORT-s | *Platycleis albopunctata* | 0 | 0 | 0 | 0 | 1 | 0 | 0 | 0 | 0 | 0 | 0 | 0 | 1 | 0 | 0 | N | 4 |
| ORT-s | *Ramburiella hispanica* | 0 | 0 | 0 | 0 | 1 | 0 | 0 | 0 | 0 | 0 | 0 | 0 | 0 | 0 | 0 | N | 2 |
| ORT-s | *Steropleurus perezi* | 1 | 0 | 0 | 0 | 1 | 0 | 1 | 2 | 0 | 1 | 0 | 0 | 0 | 3 | 0 | Y | 4 |
| ORT-s | *Tettigonia viridissima* | 0 | 1 | 0 | 1 | 0 | 0 | 0 | 1 | 2 | 0 | 0 | 0 | 0 | 0 | 0 | Y | 4 |
| ORT-s | *Thyreonotus corsicus* | 1 | 0 | 0 | 1 | 2 | 0 | 0 | 1 | 2 | 1 | 1 | 1 | 1 | 3 | 0 | Y | 4 |
| ORT-s | *Tylopsis liliifolia* | 0 | 1 | 2 | 1 | 0 | 0 | 0 | 0 | 0 | 0 | 3 | 0 | 1 | 4 | 0 | Y | 4 |
| ORT-s | *Yersinella raymondi* | 0 | 0 | 0 | 0 | 0 | 0 | 0 | 0 | 0 | 0 | 0 | 1 | 0 | 1 | 0 | N | 4 |
| REP | *Malpolon monspessulanus* | 0 | 0 | 0 | 0 | 0 | 0 | 0 | 0 | 0 | 0 | 0 | 0 | 1 | 0 | 0 | N | 1 |
| REP | *Podarcis hispanica* | 6 | 7 | 5 | 4 | 5 | 1 | 5 | 3 | 2 | 4 | 2 | 0 | 1 | 0 | 0 | Y | 1 |
| REP | *Psammodromus algirus* | 2 | 1 | 1 | 2 | 1 | 1 | 3 | 2 | 2 | 1 | 0 | 0 | 1 | 0 | 0 | Y | 1 |
| REP | *Rhinechis scalaris* | 0 | 0 | 0 | 0 | 0 | 0 | 0 | 0 | 0 | 0 | 1 | 0 | 0 | 0 | 0 | N | 1 |
| REP | *Tarentola mauritanica* | 0 | 0 | 0 | 0 | 0 | 0 | 0 | 0 | 0 | 0 | 2 | 0 | 0 | 0 | 0 | N | 1 |
| REP | *Timon Lepidus* | 1 | 0 | 0 | 0 | 0 | 0 | 0 | 1 | 0 | 0 | 1 | 0 | 0 | 0 | 0 | Y | 1 |

**Table S2**. **List of plant species and presence in the three areas**: logging (“L”), subsoiling (“S”) and unburnt (“U”).

| **SP** | **Plant type** | **“L”** | **“S”** | **“U”** |
| --- | --- | --- | --- | --- |
| *Ajuga chamaepitys* | Grass | x |  | x |
| *Allium asphaerocephalon* | Grass |  |  |  |
| *Anacamptis pyramidalis* | Grass |  |  |  |
| *Anagallis arvensis* | Grass | x | x | x |
| *Andryala integrifolia* | Grass | x |  |  |
| *Antirrhinum* sp | Grass |  | x |  |
| *Aphillanthes monspeliensis* | Grass | x | x | x |
| *Arbutus unedo* | Tree |  | x | x |
| *Argyrolobium zanonii* | Grass | x | x | x |
| *Asparagus acutifolius* | Grass |  |  | x |
| *Asperula cynanchica* | Grass | x |  |  |
| *Asphodelus cf.* | Grass |  |  |  |
| *Asteriscus spinosus* | Grass |  | x | x |
| *Asterolinon linum-stellatum* | Grass |  | x |  |
| *Astragalus monspessulanus* | Grass | x | x | x |
| *Astragalus sesameus* | Grass |  | x |  |
| *Avenula bromoides* | Grass | x |  | x |
| *Blakstonia perfoliata* | Grass | x | x | x |
| *Brachypodium distachyon* | Grass | x | x | x |
| *Brachypodium phoenicoides* | Grass | x | x | x |
| *Brachypodium retusun* | Grass | x | x | x |
| *Bupleurum fruticescens* | Grass |  | x |  |
| *Bupleurum fruticosum* | Shrub | x | x |  |
| *Campanula erinus* | Grass |  |  |  |
| *Carex flacca* | Grass | x | x | x |
| *Catapodium rigidum* | Grass | x | x |  |
| *Centaurea aspera* | Grass | x | x |  |
| *Centaurea* sp | Grass |  |  | x |
| *Centaurium pulchellum* | Grass | x | x |  |
| *Cistus albidus* | Shrub | x | x | x |
| *Cistus salvifolius* | Shrub |  | x |  |
| *Clematis flammula* | Grass |  |  | x |
| *Conopodium majus* | Grass |  |  | x |
| *Convolvulus arvensis* | Grass | x | x | x |
| *Convolvulus lineatus* | Grass |  |  |  |
| *Coriaria myrtifolia* | Shrub | x | x | x |
| *Coris monspeliensis* | Grass | x | x | x |
| *Crupina* sp | Grass | x |  |  |
| *Crupina vulgaris* | Grass |  |  |  |
| *Dactylis glomerata* | Grass |  | x | x |
| *Daphne gnidium* | Shrub | x | x | x |
| *Dorycnium hirsutum* | Grass | x | x | x |
| *Dorycnium pentaphyllum* | Grass | x | x | x |
| *Echium vulgare* | Grass | x | x |  |
| *Epipactis cf. atrorubens* | Grass |  |  | x |
| *Erica multiflora* | Shrub | x | x | x |
| *Erucastrum cf.* | Grass |  |  |  |
| *Erucastrum nasturtiifolium* | Grass |  | x |  |
| *Eryngium campestre* | Grass | x | x | x |
| *Euphorbia cf. exigua* | Grass |  |  | x |
| *Euphorbia nicaeensis* | Grass |  |  | x |
| *Euphorbia serrata* | Grass | x | x | x |
| *Euphorbia* sp | Grass |  | x |  |
| *Festuca gr. ovina* | Grass | x |  |  |
| *Filago pyramidata* | Grass | x | x |  |
| *Fumana ericoides* | Grass | x | x | x |
| *Galactites tomentosa* | Grass |  | x |  |
| *Galium cf. lucidum* | Grass | x |  |  |
| *Galium pumilum* | Grass |  | x |  |
| *Genista scorpius* | Shrub | x | x | x |
| *Globularia alypum* | Shrub | x |  |  |
| *Helianthemum oelandicum* | Grass | x | x | x |
| *Helichrysum stoechas* | Grass | x | x | x |
| *Hieracium* sp | Grass | x | x | x |
| *Hippocrepis comosa* | Grass | x | x | x |
| *Hypericum perforatum* | Grass |  |  | x |
| *Hypochoeris radicata* | Grass | x |  |  |
| *Juniperus oxycedrus* | Shrub | x | x | x |
| *Koeleria vallesiana* | Grass |  |  | x |
| *Lactuca serriola* | Grass | x | x |  |
| *Lavandula latifolia* | Grass |  |  | x |
| *Leontodon taraxacoides* | Grass | x | x | x |
| *Leuzea conifer* | Grass | x |  |  |
| *Ligustrum vulgare* | Tree |  |  | x |
| *Linum narbonense* | Grass |  | x |  |
| *Linum strictum* | Grass | x |  |  |
| *Linum tenuifolium* | Grass | x | x | x |
| *Lonicera implexa* | Shrub | x | x | x |
| *Medicago lupulina* | Grass | x | x | x |
| *Medicago minima* | Grass | x | x |  |
| *Medicago* sp | Grass | x |  | x |
| *Melilotus* sp | Grass | x | x | x |
| *Minuartia cf.* | Grass | x |  |  |
| *Olea europaea* | Tree |  |  | x |
| *Onobrychis saxatilis* | Grass | x | x | x |
| *Ononis minutissima* | Shrub | x | x | x |
| *Ononis natrix* | Shrub | x |  |  |
| *Orobanche* sp | Grass | x | x | x |
| *Oryzopsis miliacea* | Grass | x |  |  |
| *Phagnalon rupestre* | Grass | x |  |  |
| *Phleum phleoides* | Grass |  | x |  |
| *Pinus* sp | Tree | x | x | x |
| *Pistacia lentiscus* | Shrub | x | x | x |
| *Plantago lanceolata* | Grass | x | x | x |
| *Polygala rupestris* | Grass | x | x | x |
| *Populus alba* | Tree | x |  |  |
| *Populus nigra* | Tree | x |  |  |
| *Psoralea bituminosa* | Grass | x | x | x |
| *Quercus coccifera* | Shrub | x |  |  |
| *Quercus ilex* | Tree | x | x | x |
| *Quercus x cerrioides* | Tree |  | x | x |
| *Reseda phyteuma* | Grass |  | x |  |
| *Rhamnus alaternus* | Shrub |  |  | x |
| *Rosa* sp | Shrub |  | x |  |
| *Rosmarinus officinalis* | Shrub | x | x | x |
| *Rubia peregrine* | Grass | x | x | x |
| *Rubus ulmifolius* | Shrub |  | x | x |
| *Sanguisorba minor* | Grass | x | x | x |
| *Santolina chamaecyparissus* | Grass |  |  | x |
| *Satureja calamintha* | Grass |  |  | x |
| *Satureja Montana* | Grass |  |  | x |
| *Scorzonera angustifolia* | Grass | x | x | x |
| *Sedum sediforme* | Grass | x | x |  |
| *Sideritis hirsute* | Grass |  | x |  |
| *Smilax aspera* | Shrub |  |  | x |
| *Sonchus asper* | Grass |  |  |  |
| *Sonchus* sp | Grass | x |  | x |
| *Sonchus tenerrimus* | Grass | x | x | x |
| *Spartium junceum* | Shrub |  |  |  |
| *Staehelina dubia* | Grass |  |  | x |
| *Stipa cf. Offneri* | Grass | x |  | x |
| *Sylibum marianum* | Grass | x |  |  |
| *Teucrium botrys* | Grass | x | x |  |
| *Teucrium chamaedrys* | Grass | x | x | x |
| *Teucrium polium* | Grass |  | x | x |
| *Thymus vulgaris* | Grass | x | x | x |
| *Trigonella monspeliaca* | Grass | x |  |  |
| *Ulex parviflorus* | Shrub |  | x |  |
| *Urospermum picroides* | Grass |  |  | x |
| *Verbascum* sp | Grass |  | x |  |
| *Verbena officinalis* | Grass | x | x |  |
| *Vinca* sp | Grass |  |  | x |
| *Viola* sp | Grass | x | x |  |
| *Vitis* sp | Shrub | x |  | x |

**Table S3. R values and significance (* denotes p < 0.05) from the ANOSIM taxonomic analysis of each animal group.** The last rows are R values for the overall animal (abundance) and plant (presence/absence) species. The Global R column indicates the overall comparison of the three areas. The rest of the columns indicate the pairwise comparison between areas, with the R value and significance. Acronyms of the three areas are unburnt reference (“U”), logging (“L”) and subsoiling (“S”). For acronyms of groups, see text.

| Taxonomic  Groups | Global R | “U”-”L” | “U”-”S” | “L”-”S” |
| --- | --- | --- | --- | --- |
| GAS SOIL | 0.555* | 0.636* | 0.872* | 0.128 |
| FOR SOIL | 0.207* | 0.196 | 0.212 | 0.220 |
| COL SOIL | 0.172 | 0.382* | 0.262 | -0.194 |
| ARA SOIL | 0.342* | 0.364* | 0.614* | 0.080 |
| HETE SOIL | 0.1720* | 0.167 | 0.3680* | -0.004 |
| FOR VEG | 0.163 | 0.324* | 0.220 | -0.100 |
| ORT VEG | 0.018 | -0.054 | 0.068 | 0.006 |
| COL VEG | 0.526* | 0.820* | 0.812* | 0.008 |
| REP | 0.344* | 0.471* | 0.336* | 0.128 |
| AVES | 0.694* | 0.8380* | 0.9920* | 0.1700 |
| Total Animals | 0.708* | 0.896* | 0.916* | 0.348* |
| Total Plants | 0.414* | 0.586* | 0.530* | 0.120 |

**Table S4.** **R values and significance (* denotes p < 0.05) from the ANOSIM functional (dietary) analysis of each animal group.** The Global R column indicates the overall comparison among the three areas. The rest of the columns indicate the pairwise comparison between areas, with the R value and signification. Acronyms of the three areas are unburnt reference (“U”), logging (“L”) and subsoiling (“S”). For acronyms of groups, see text.

|  | Global R | “U”-”L” | “U”-”S” | “L”-”S” |
| --- | --- | --- | --- | --- |
| GAS SOIL | 0.164 | -0.064 | 0.472* | 0.108 |
| ARA SOIL | 0.674* | 0.484* | 0.568* | 0.982* |
| HETE SOIL | 0.0370 | 0.121 | 0.065 | -0.07 |
| FOR SOIL | 0.001 | 0.044 | 0 | -0.036 |
| FOR VEG | 0.133 | 0.108 | 0.268 | 0.018 |
| COL SOIL | 0.184 | 0.188 | 0.452* | -0.112 |
| COL VEG | 0.372* | 0.732* | 0.496* | -0.152 |
| ORT VEG | 0.045 | 0.202 | 0.018 | -0.028 |
| REP | 0.200 | 0.327 | 0.188 | 0.042 |
| AVES | 0.594* | 0.746* | 0.712* | 0.300* |
| Total Fauna | 0.414* | 0.488* | 0.648* | 0.144 |

**Figure S1.** **Comparison of the total number of animal species and evenness among areas for each animal group:** unburnt “U”, logging “L” and subsoiling “S”. Each column represents average scores ± standard error. Each figure includes the ANOVA or Kruskall-Wallis tests, and letters refer to post hoc comparisons between areas.
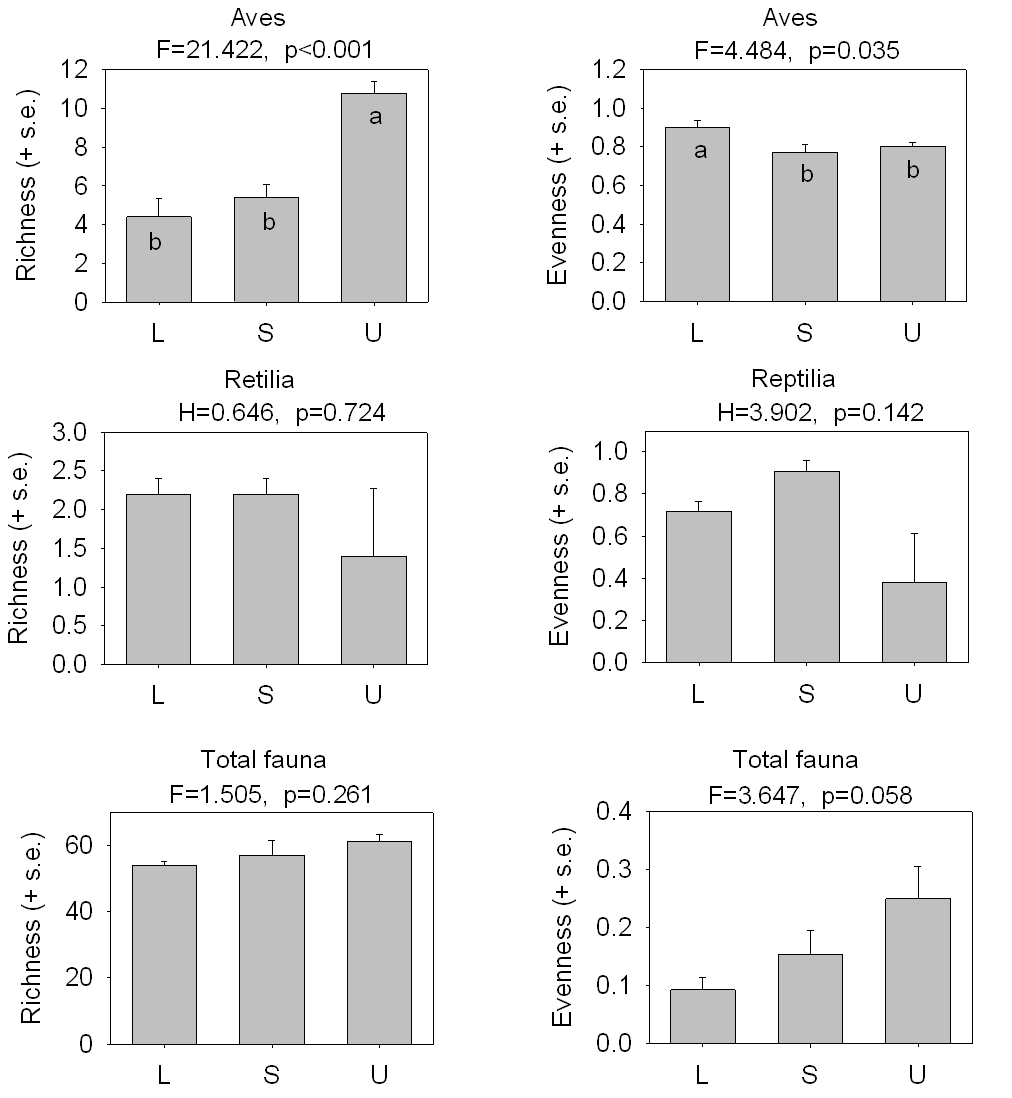


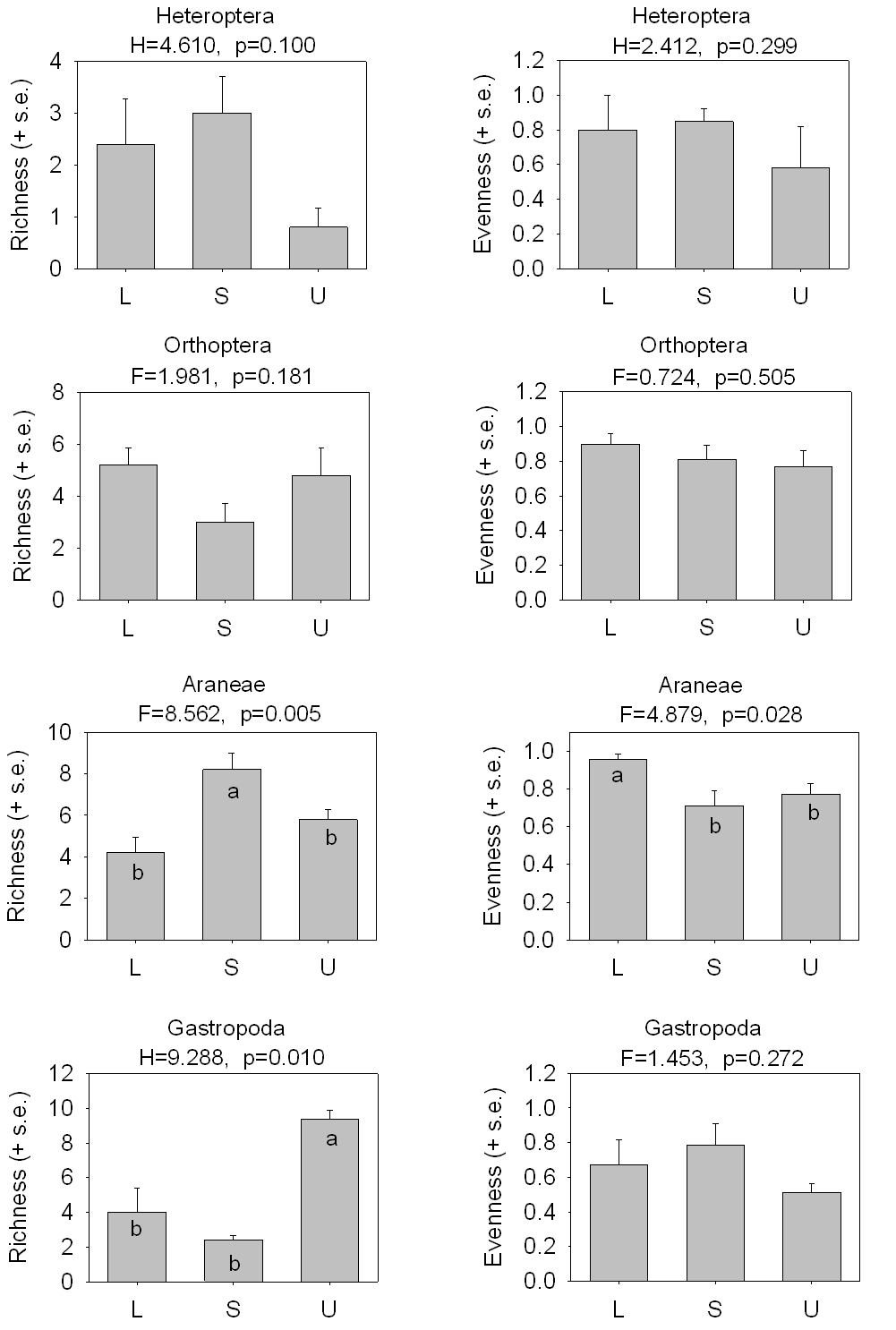


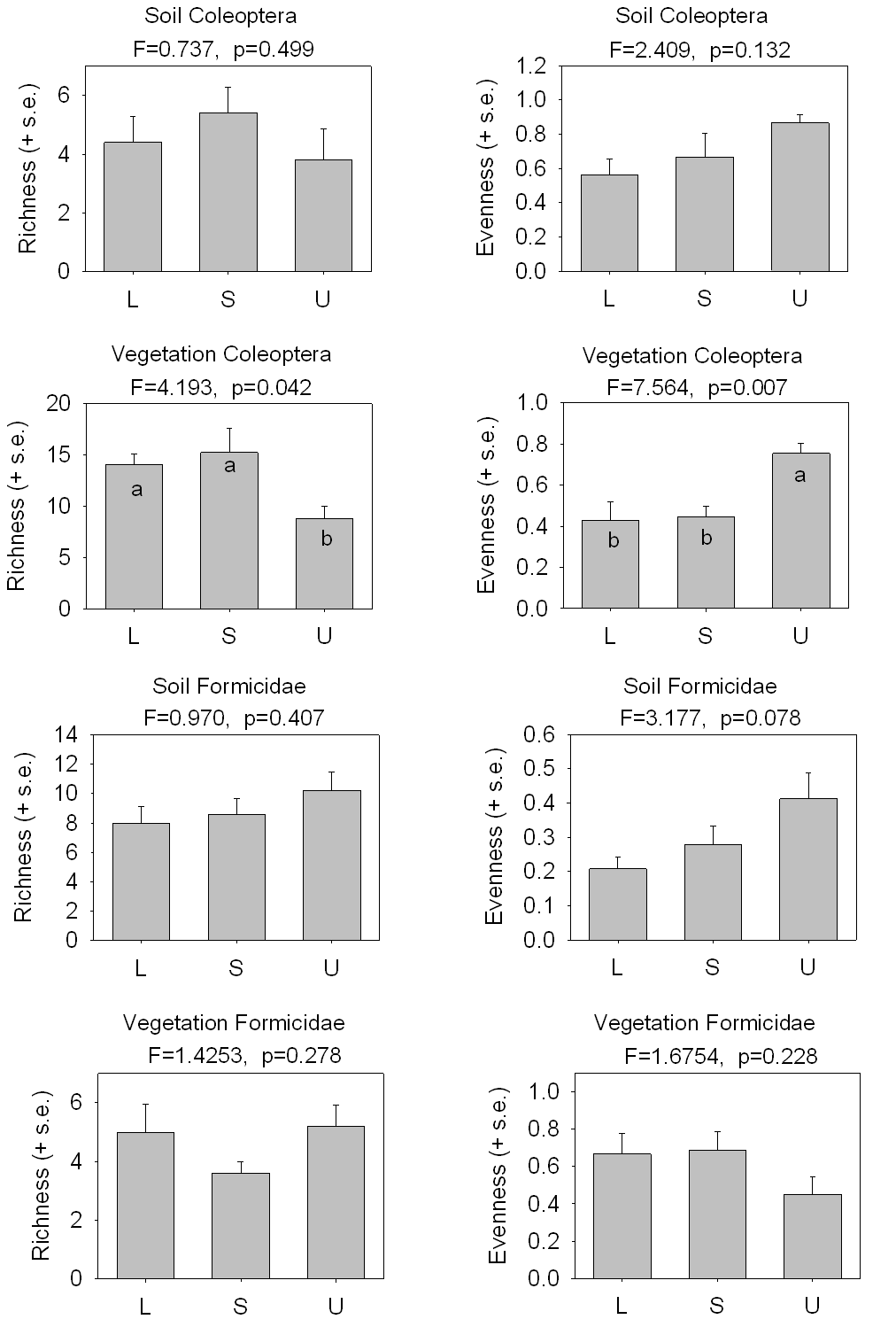

Supplement: File S1 — Supporting information file including Text S1, Tables S1–S4, and Figure S1. Text S1. Description of the sampling methods. Table S1. Basic data of the 15 sampled plots and abundances of animal species: burnt logging (L1 to L5), burnt subsoiling (S1 to S5) and unburnt (U1 to U5); Data: species included (Y) and excluded (N) in statistical analyses according to the number of records. FG (feeding groups): zoophagous (1), phytophagous (2), saprovorous (3) and omnivorous (4). Table S2. List of plant species and presence in the three areas: logging (“L”), subsoiling (“S”) and unburnt (“U”). Table S3. R values and significance (* denotes p<0.05) from the ANOSIM taxonomic analysis of each animal group. The last rows are R values for the overall animal (abundance) and plant (presence/absence) species. The Global R column indicates the overall comparison of the three areas. The rest of the columns indicate the pairwise comparison between areas, with the R value and significance. Acronyms of the three areas are unburnt reference (“U”), logging (“L”) and subsoiling (“S”). For acronyms of groups, see text. Table S4. R values and significance (* denotes p<0.05) from the ANOSIM functional (dietary) analysis of each animal group. The Global R column indicates the overall comparison among the three areas. The rest of the columns indicate the pairwise comparison between areas, with the R value and signification. Acronyms of the three areas are unburnt reference (“U”), logging (“L”) and subsoiling (“S”). For acronyms of groups, see text. Figure S1. Comparison of the total number of animal species and evenness among areas for each animal group: unburnt “U”, logging “L” and subsoiling “S”. Each column represents average scores ± standard error. Each figure includes the ANOVA or Kruskall-Wallis tests, and letters refer to post hoc comparisons between areas. (DOCX) [file pone.0088224.s001.docx]
